# Assessing the Anti-inflammatory Mechanism of Reduning Injection by Network Pharmacology

**DOI:** 10.1155/2020/6134098

**Published:** 2020-12-16

**Authors:** Fuda Xie, Mingxiang Xie, Yibing Yang, Miaomiao Zhang, Xiaojie Xu, Na Liu, Wei Xiao, Jiangyong Gu

**Affiliations:** ^1^Research Center of Integrative Medicine, School of Basic Medical Science, Guangzhou University of Chinese Medicine, Guangzhou 510006, China; ^2^Department of Biochemistry, School of Basic Medical Science, Guangzhou University of Chinese Medicine, Guangzhou 510006, China; ^3^The Second Clinical College, Guangzhou University of Chinese Medicine, Guangzhou 510006, China; ^4^College of Chemistry and Molecular Engineering, Peking University, Beijing 100871, China; ^5^State Key Laboratory of New-Tech for Chinese Medicine Pharmaceutical Process, Lianyungang 222001, China

## Abstract

Reduning Injection (RDNI) is a traditional Chinese medicine formula indicated for the treatment of inflammatory diseases. However, the molecular mechanism of RDNI is unclear. The information of RDNI ingredients was collected from previous studies. Targets of them were obtained by data mining and molecular docking. The information of targets and related pathways was collected in UniProt and KEGG. Networks were constructed and analyzed by Cytoscape to identify key compounds, targets, and pathways. Data mining and molecular docking identified 11 compounds, 84 targets, and 201 pathways that are related to the anti-inflammatory activity of RDNI. Network analysis identified two key compounds (caffeic acid and ferulic acid), five key targets (Bcl-2, eNOS, PTGS2, PPARA, and MMPs), and four key pathways (estrogen signaling pathway, PI3K-AKT signaling pathway, cGMP-PKG signaling pathway, and calcium signaling pathway) which would play critical roles in the treatment of inflammatory diseases by RDNI. The cross-talks among pathways provided a deeper understanding of anti-inflammatory effect of RDNI. RDNI is capable of regulating multiple biological processes and treating inflammation at a systems level. Network pharmacology is a practical approach to explore the therapeutic mechanism of TCM for complex disease.

## 1. Introduction

Inflammation is regarded as a kind of congenital immunity as well as the basis of various physiological and pathological processes, and it can affect human health and living quality in many respects [1–4]. Five typical symptoms of inflammation are fever, pain, redness, swelling, and loss of function [5]. Acute inflammation is the body's initial response to harmful stimuli such as burns, pathogen infection, and toxins [6, 7]. Chronic inflammation is a biological progress that leads to multiple diseases such as hay fever, periodontitis, atherosclerosis, rheumatoid arthritis, and even cancer [8–10]. There are at present more than one hundred FDA-approved anti-inflammatory drugs, which are classified into steroid and nonsteroidal anti-inflammatory drugs [11–13]. However, these drugs are related to various side effects including irreversible sensor neural hearing loss [14], gastrointestinal symptoms (such as dyspepsia, gastrointestinal bleeds, or even gastrointestinal perforations) [15], and side effects on cartilage metabolism. [16]

Traditional Chinese medicines (TCMs) have been used to treat various diseases including inflammation for a long time. For example, Reduning Injection [17], Bi-Qi capsule [18], and Shuanghuanglian injection [19] are widely used in China for treating inflammation. The validity and safety of these TCM formulas are already verified by thousands of years of clinical applications. Some of them have been studied in modern approaches, and their effectiveness and molecular mechanism were demonstrated by the results [17, 20, 21]. TCM can regulate multiple pathogenic progresses so as to cure diseases effectively and completely in a holistic manner [19, 20, 22]. However, the ingredients of TCM are complicated and can interact with multiple targets. It is difficult to elucidate the mechanism of the action (MOA) of TCM by traditional pharmacological methods.

Network pharmacology provides frameworks to understand how regulation arises from the interactions between cellular components, and it is considered the next paradigm in drug development [23]. By using network pharmacology approaches, we can build complex networks on the basis of disease-related biological progresses and adopt network analysis to obtain insights into the pharmacological mechanism. These methods can provide theoretical basis and guidance for the development of multitarget drugs, and they have been used to investigate the pathogenesis of several diseases [22, 24, 25]. Reduning Injection (RDNI) is used for the treatment of inflammatory diseases, such as upper respiratory tract infection and acute bronchitis [26–28], while the molecular mechanism of its therapeutic function is unclear. Three herbs contained in RDNI are wildly used to cure inflammation-related diseases, namely, *Lonicera japonica* Thunb. (honeysuckle, Jinyinhua), *Gardenia jasminoides* Ellis. (cape jasmine, Zhizi), and *Artemisia annua* L. (sweet wormwood, Qinghao) [29–31]. In our previous works, the main ingredients of RDNI and their activities against inflammation have been explored [17, 22, 27]. In this work, data mining and molecular docking were used to predict the targets of RDNI compounds and their metabolites. A compound-target network and a target-pathway network were constructed. Key targets and pathways were identified by network analysis and literature consulting. The cross-talks between inflammation-related pathways were also discussed. The results indicate that the molecular mechanism of the anti-inflammatory function of RDNI can be discovered by computational modeling, which provides a practical approach to study the MOA of TCM prescription.

## 2. Methods

### 2.1. Collection of RDNI Compounds

Nine ingredients with measurable content of RDNI have been identified in previous works [32–38]. Four metabolites of these ingredients were gathered by literature mining [39–41]. The information and 3D structures of these 13 compounds were downloaded from PubChem (http://pubchem.ncbi.nlm.nih.gov) [42], a chemical database of authoritative sources.

### 2.2. Target Mining

The targets of RDNI compounds and metabolites were collected by database searching and molecular docking. Four databases were used in this step, namely, PubChem, Traditional Chinese Medicine Systems Pharmacology Database and Analysis Platform (TCMSP, http://lsp.nwu.edu.cn/tcmsp.php) [43], Binding DataBase (BindingDB, http://www.bindingdb.org/bind/index.jsp) [44, 45], and DrugBank (http://www.drugbank.ca) [46]. TCMSP is a systems pharmacology platform that provides the relationships between Chinese herbal medicines and their targets. BindingDB can provide measured binding affinities between compounds and their targets. First, CID codes in PubChem were used to find the records of RDNI compounds, and their targets were obtained in the “Biological Test Results.” Second, the records of compounds in TCMSP were retrieved by the CAS registry number, and the targets' information was collected from the “Related Targets” section. Finally, the tool “Find my Compound's Target” in BindingDB was used to screen targets of RDNI compounds. Targets gathered in PubChem, BindingDB, and TCMSP are recorded in Supplementary Table S1.

The DrugBank database can provide detailed drug data and comprehensive drug target information. There are 126 FDA-approved anti-inflammatory drugs in DrugBank [46]. Their targets were collected, and known protein structures were downloaded from the RSCB protein data bank (http://www.rcsb.org). Molecular docking was adopted to evaluate the binding affinity between each compound and target by Autodock 4.2.6 [47]. The energy grid was a 30 × 30 × 30 Å cube centered on the occupied space of the original ligand with a spacing of 0.375 Å between the grid points. The Lamarckian genetic algorithm (LGA) was used to optimize the conformation of compound in the binding pocket. The parameters for LGA were listed as follows: the number of individuals in population, maximum number of energy evaluations, and the maximum number of generations, and the rate of gene mutation was set as 150, 2.5 × 10^6^, 2.7 × 10^4^, and 0.02, respectively. Other parameters were set to default. The docking results were sorted according to the binding energy, and the proteins with binding energy lower than -8.18 kcal/mol (the threshold for inhibition constant was 1 *μ*M) were regarded as a target of the corresponding compound.

### 2.3. Network Construction

On the basis of compound-target interaction obtained in the previous step, the compound-target network (CTN) was constructed and visualized by Cytoscape version 3.6.1 [48]. Key targets and main active components of RDNI for treating inflammation were predicted by degree centrality, betweenness centrality, and closeness centrality. These network topological parameters were calculated by the NetworkAnalyzer plugin [49]. Targets that meet two screening criteria were regarded as important targets: their degree centralities were in the top ten of all involved targets; their betweenness centralities and closeness centralities were both larger than the average value of all involved targets in CTN (Supplementary Table S2). Key targets were selected from these important targets after assessment of their locations in different pathways and their regulation relationships with upstream and downstream targets. The molecular functions and related biological processes of targets were retrieved from UniProt (http://www.uniprot.org) [50].

The related pathways (Supplementary Table S3) of RDNI targets were collected from the Kyoto Encyclopedia of Genes and Genomes (KEGG, http://www.kegg.jp) [51]. The target-pathway network (TPN) was then constructed and visualized by Cytoscape. Key pathways of RDNI were obtained on the basis of TPN and literature consulting. Twelve pathways were found to have close relationships with inflammation, and they also had high degree centralities (>6) as well as high betweenness centralities (>average value) in the TPN. Four of the twelve pathways were excluded because they do not represent a specific biological process. Another two pathways were excluded because they do not have high closeness centralities (>average value) (Supplementary Table S4). The remaining six pathways were regarded as important pathways of RDNI, and a compound-target-pathway network (CTPN) was constructed by Cytoscape. Four key pathways were selected from important pathways for their close relationships with inflammatory processes. Graphs of key pathways were downloaded from KEGG. Targets of RDNI were marked in red by KEGG mapping tools.

A cross-talk network was constructed to visualize the cross-talks among key pathways and other three pathways, which were closely associated with both the four key pathways and inflammatory processes.

## 3. Results and Discussion

### 3.1. Analysis of Compound-Target Network

**Figure 1:**
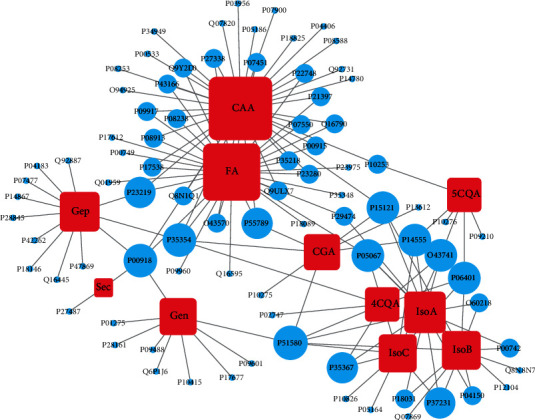
Compound-target network of RDNI. Red squares represent compounds and their metabolites of RDNI, and blue ellipses represent targets. The interaction between compound and target is represented by gray edges. The size of nodes varies with the degree centrality in this network.

The CAS entry, PubChem CID, molecular weight, and content in RDNI of the RDNI compounds are recorded in Table 1. Sixty-seven known targets for 9 compounds and 2 metabolites of RDNI were found in PubChem, BindingDB, and TCMSP (Supplementary Table S1). One hundred and seventeen targets are associated with FDA-approved anti-inflammatory drugs according to DrugBank. Sixty-four targets have 3D structures and can be utilized for molecular docking. Nineteen targets were obtained by molecular docking (Table 2). In total, eighty-four targets were obtained after integration and deduplication. The compound-target network (Figure 1) contains 95 nodes (11 compounds and 84 targets) and 144 edges. Edges in this network reflect the diverse regulations of RDNI for inflammation-related processes.

These compounds and their metabolites have complex interactions with cellular targets. First, caffeic acid (CAA) and its metabolite ferulic acid (FA) have wide influences on inflammatory processes. CAA has 36 targets, and FA has 32 targets, with 20 targets in common. Many targets are related to the regulation of inflammatory process such as arachidonate 5-lipoxygenase (ALOX5, UniProt: P09917), heat shock protein HSP 90-alpha (HSP90AA1, UniProt: P07900), and prostaglandin G/H synthase 2 (PTGS2, UniProt: P35354). Second, isochlorogenic acid A (IsoA), isochlorogenic acid B (IsoB), and isochlorogenic acid C (IsoC) have 13, 11, and 9 targets, respectively. Most proteins have explicit relationship with inflammation because they are targets of FDA-approved anti-inflammatory drugs. Geniposide (Gen) and its metabolite genipin (Gep) have 12 and 9 targets, respectively, while only one target has affinity with both of them. Third, secoxyloganin (Sec), chlorogenic acid (CGA), cryptochlorogenic acid (4CQA), and neochlorogenic acid (5CQA) have 2, 4, 6, and 6 targets, respectively. They are also important to the therapeutic effect of RDNI as the high content.

Eight targets, namely, thiopurine S-methyl transferase (TPMT, UniProt: P51580), carbonic anhydrase 2 (CA2, UniProt: P00918), amyloid-beta A4 protein (APP, UniProt: P05067), progesterone receptor (PGR, UniProt: P06401), membrane associated phospholipase A2 (PLA2G2A, UniProt: P14555), 5′-AMP-activated protein kinase subunit beta-2 (PRKAB2, UniProt: O43741), aldose reductase (AKR1B1, UniProt: P15121), and PTGS2 would bind with more than 3 compounds of RDNI. Four proteins, namely, histamine H1 receptor (HRH1, UniProt: P35367), peroxisome proliferator-activated receptor gamma (PPARG, UniProt: P37231), prostaglandin G/H synthase 1 (PTGS1, UniProt: P23219), and FAD-linked sulfhydryl oxidase ALR (GFER, UniProt: P55789), can be targeted by 3 compounds of RDNI. These top twelve targets screened by degree centrality have high betweenness centralities (>average value). Seven of them have high closeness centralities (>average value). Other targets can interact with one or two compounds of RDNI. Many of these targets would play significant roles in regulating inflammatory processes.

For example, PTGS2 is the target of CGA, 4CQA, CAA, FA, and Gep. It is the major enzyme responsible for the production of inflammatory prostaglandins and expresses in the inflammatory process only [17]. First, the prostaglandin E2 (PGE2) synthesized by PTGS2 can promote the production of multifarious inflammatory factors, such as interleukin-10 (IL-10, UniProt: P22301) [52], interleukin-8 (IL-8, UniProt: P10145), and tumor necrosis factor alpha (TNF*α*, UniProt: P01375) [53]. Second, PGE2 can regulate the production of adenosine 3′,5′-cyclic phosphate (cAMP) and the expressions of inflammatory genes which are regulated by this second messenger [54]. Third, PGE2 is also responsible for some inflammatory symptoms, such as cough [55] and airflow obstruction, because it can regulate the production of mucin in the respiratory tract [56]. Fourth, PTGS2 can influence inflammatory targets such as 72 kDa type IV collagenase (MMP2, UniProt: P08253), matrix metalloproteinase 9 (MMP9, UniProt: P14780), and nitric-oxide synthase, endothelial (eNOS, UniProt: P29474) [57, 58]. Finally, PTGS2 is also the key regulatory factor of differentiation of T helper cell 17 (Th17) in inflammatory processes [59].

Apoptosis regulator Bcl-2 (Bcl-2, UniProt: P10415) is the target of Gen. After binding with Bcl-2-like protein 1 (Bcl-xl, UniProt: P07817), Bcl-2 can regulate the expression of caspase 1 (CASP1, UniProt: P29466) by inhibiting NLR family protein LRR and PYD domain-containing protein 1 (NALP1, UniProt: Q9C00). CASP1 participates in the dissociation of substrates involved in cell apoptosis and inflammation, and it is capable of catalyzing the maturation and secretion of interleukin-1 beta (IL-1*β*, UniProt: P01584) [60, 61]. NALP1 participates in the process of inflammation by activating the secretion of high mobility group protein B1 (HMGB1, UniProt: P09429). Bcl-2 is also associated with particulate matter-induced pneumonia and allergic airway inflammation by regulating the apoptosis process of inflammatory cells [62, 63].

FA and IsoA can bind with eNOS and then regulate the production of nitric oxide (NO), which plays an important role in inflammatory processes [64]. The overproduction of NO participates in inflammatory response by regulating a lot of biological processes, such as the synthesis of iron-nitrite complex, the inhibition of DNA ligase, the promotion of plasma exudation, and edema formation [65, 66]. The protective effect of eNOS against systemic inflammation is also proved [67].

Peroxisome proliferator-activated receptor alpha (PPARA, UniProt: Q07869) is a member of nuclear hormone receptor superfamily and a target of IsoB. It participates in the regulation of lipid metabolism, adipocyte differentiation, and inflammatory process by activating related transcription factors [68]. Interstitial collagenase (MMP1, UniProt: P03956), MMP2, and MMP9 are both targets of FA and IsoA. MMPs can mediate the pretreatment of TNF*α* and therefore participate in regulation of inflammatory processes [69]. Induced myeloid leukemia cell differentiation protein Mcl-1 (Mcl-1, UniProt: Q07820) is a target of CAA. It is involved in cell survival, cell apoptosis, and inflammation [70]. Therefore, compounds of RDNI can regulate the inflammatory process through complex interactions with inflammation-associated targets.

### 3.2. Analysis of Target-Pathway Network

**Figure 2:**
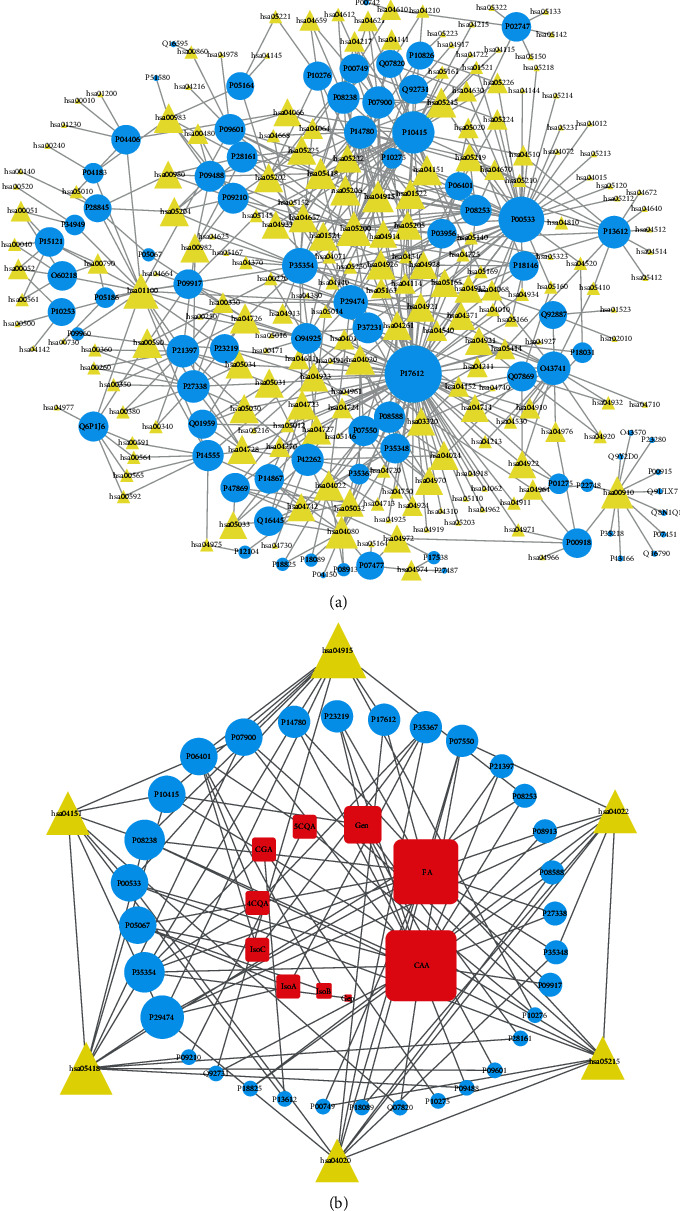
Target-pathway network (a) and compound-target-pathway network (b). Red squares, blue ellipses, and yellow triangles represent small compounds, targets, and pathways, respectively. Gray edges correspond to the relationships between compounds, targets, and pathways. The size of a node is directly proportional to degree centrality.

Eighty targets of RDNI are involved in 201 pathways according to KEGG (Supplementary Table S2). The target-pathway network (Figure 2(a)) contains 281 nodes (80 targets and 201 pathways) and 577 edges. The TPN shows the complexity and diversity of regulatory effects of RDNI on human biological processes. Twelve pathways would have close relationship with inflammation since they have large degree centrality (>6). Six pathways are excluded because they do not represent a specific biological process or do not have high closeness centralities, namely, metabolic pathways, pathways in cancer, neuroactive ligand-receptor interaction, nitrogen metabolism, serotonergic synapse, and microRNAs in cancer. The rest 6 pathways constitute the compound-target-pathway network (Figure 2(b)) by linking 32 targets and 10 compounds. The CTPN contains 48 nodes and 102 edges. Figures 2(a) and 2(b) reflect the multiple regulatory functions of RDNI through different pathways. Four of them were found to have high correlation with the regulation of inflammatory response, namely, estrogen signaling pathway (hsa04915), PI3K-AKT signaling pathway (hsa04151), cGMP-PKG signaling pathway (hsa04022), and calcium signaling pathway (hsa04020).

**Figure 3:**
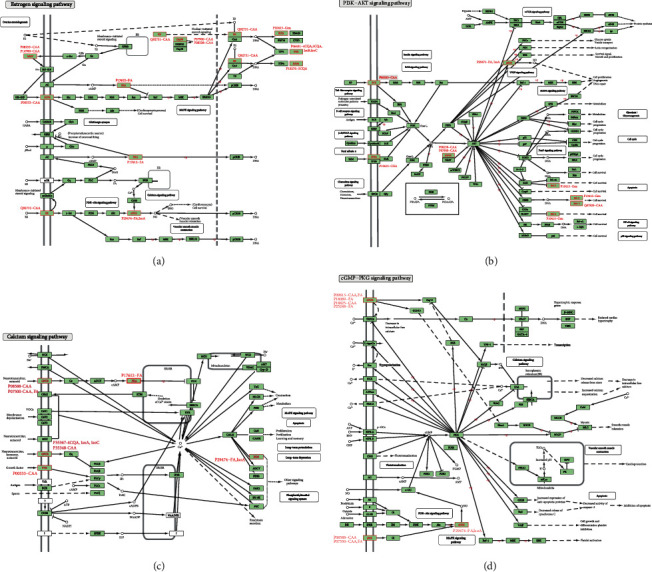
Diagram of the estrogen signaling pathway (a), PI3K-AKT signaling pathway (b), calcium signaling pathway (c), and cGMP-PKG signaling pathway (d). Names of each pathway are labeled in the upper left corner. The origin pathway images are downloaded from KEGG. The targets of RDNI are marked in red. UniProt ID of a specific target and the compound(s) binding with it are labeled nearby. Permission is granted by Kanehisa Laboratories to use these KEGG pathway map images.

The estrogen signaling pathway (Figure 3(a)) contains the most targets of RDNI than other pathways. It regulates many physiological processes such as reproduction, cardiovascular protection, cellular homeostasis, and inflammatory metabolic process [71–73]. Eight compounds (CAA, FA, 4CQA, 5CQA, IsoA, IsoB, IsoC, and Gen) can interact with eleven targets (P00533, P06401, P07900, P08238, P08253, P10276, P10415, P14780, P17612, P29474, and Q92731) in this pathway. First, CAA can regulate the estrogen signaling pathway by binding with HSP90 and modulate estrogen receptor beta (ESR2, UniProt: Q92731). Second, HSP90 is involved in the formation process of the complex of estrogen receptor (ER) and nuclear receptor coactivator (CoA). The ER-CoA complex can activate the expression of ER-dependent genes, which participate in the regulation of cell apoptosis and inflammation, such as Bcl-2, PGR, and retinoic acid receptor alpha (RARA, UniProt: P10276). Third, MMP2, MMP9, ER, and epidermal growth factor receptor (EGFR, UniProt: P00533) can regulate second messengers that play important roles in other inflammation-related pathways, such as cAMP, Ca^2+^, and phosphatidylinositol-3,4,5-trisphosphate (PIP_3_). The compounds of RDNI can regulate the inflammatory process by regulating the estrogen signaling pathway in many ways.

The PI3K-AKT signaling pathway (Figure 3(b)) is responsible for multiple fundamental cellular functions by the phosphorylation of serine/threonine kinase (AKT) [74–76]. It can also regulate inflammatory processes in many approaches [77]. Five RDNI compounds (CAA, FA, CGA, IsoA, and Gen) have influences on seven targets of this pathway (P00533, P07900, P08238, P10415, P13612, P29474, and Q07820). CAA can inhibit EGFR and then regulate the production of PIP_3_. PIP_3_, as well as the complex of HSP90 and Hsp90 cochaperone Cdc37 (UniProt: Q16543), can activate AKT [78, 79]. Activation of AKT leads to phosphorylation of downstream targets which are associated with the inflammatory process. For example, the phosphorylation of inhibitor of nuclear factor kappa-B kinase (IKK) promotes the dissociation of the complex of NF-kappa-B inhibitor alpha (I*κ*B*α*) and nuclear factor kappa-B (NF-*κ*B), and then, NF-*κ*B is released [80]. The phosphorylation of eNOS has an impact on the production of NO in the body. The phosphorylation of cyclic AMP-responsive element-binding protein (CREB) activates the expressions of Bcl-2 and Mcl-1. The phosphorylation of Bcl2-associated agonist of cell death (BAD, UniProt: Q92934) inhibits the expression of Bcl-2 and Bcl-xl. The phosphorylation of the complex of retinoic acid receptor RXR-alpha (RXRA, UniProt: P19793) and nuclear receptor subfamily 4 group A member 1 (NUR77) inhibits the expression of Bcl-2. Compounds of RDNI, such as FA, IsoA, and Gen, can also regulate these inflammation-related targets directly.

The calcium signaling pathway (Figure 3(c)) maintains the equilibrium of calcium concentration in the body and therefore mediates signal transduction in cellular and physiological processes [81, 82]. Five compounds of RDNI (CAA, FA, IsoA, IsoC, and 4CQA) would bind with seven targets of this pathway (P00533, P07550, P08588, P17612, P29474, P35348, and P35367). The impact of these compounds on the calcium signaling pathway is reflected in the regulation of phospholipase C (PLC). First, CAA regulates PLC*γ* by inhibiting EGFR in the calcium signaling pathway. Second, HRH1, a subtype of G-protein coupled receptor (GPCR), is the target of FA, IsoA, 4CQA, and IsoC and activates PLC*β*. Third, cAMP is the activator of PLC*ε* and also regulates PLC*δ* by regulating Ca^2+^ concentration. PLC produces D-myo-inositol 1,4,5-trisphosphate (IP3) and diacylglycerol (DAG); then, IP3 and DAG activate protein kinase C (PKC). PKC can regulate lipopolysaccharide-induced macrophage functions involved in inflammation [83]. It can also participate in inflammatory response by regulating NF-*κ*B-induced gene expression through the IL-1*α*-dependent induction of I*κ*B*α* [84].

The cGMP-PKG signaling pathway (Figure 3(d)) regulates a broad array of physiologic processes, such as vascular smooth muscle contraction, cell apoptosis, and inflammation [85]. The regulatory function is implemented through the phosphorylation function of cGMP-dependent protein kinase (PKG), a downstream protein of 3′,5′-cyclic GMP (cGMP) [86, 87]. Three RDNI compounds (CAA, FA, and IsoA) can interact with seven targets of this pathway (P07550, P08588, P08913, P18089, P18825, P29474, and P35348). CAA and FA can interact with alpha adrenergic receptors (*α*ARs, P08913, P18089, P18825, and P35348) and beta adrenergic receptors (*β*ARs, P07550, and P08588). *α*ARs activate guanine nucleotide-binding protein subunit alpha-11 (GNA11, UniProt: P29992) and guanine nucleotide-binding protein G(q) subunit alpha (GNAQ, UniProt: P50148). GAN11 and GANQ can further mediate the generation of Ca^2+^ by activating IP3R. *β*ARs would activate guanine nucleotide-binding protein G(i) subunit alpha (Gi). Gi can conduct the stimulus signal to eNOS and then mediate the generation of NO, while FA and IsoA can bind with eNOS directly. NO regulates the generation of cGMP by activating s-GC. cGMP can activate PKG which regulates multitudinous targets, including inflammation-related targets (CREB, BAD) and targets responsible for Ca^2+^ concentrate in the body, such as PLC*β*, protein MRVI1 (UniProt: Q9Y6F6), and cardiac phospholamban (PLB, UniProt: P26678).

The other two pathways are fluid shear stress and atherosclerosis pathway (hsa05418) and prostate cancer pathway (hsa05215). The fluid shear stress and atherosclerosis pathway regulates the progress of atherosclerosis and correlates with the activation of proinflammatory gene expression as well as early atherogenic inflammation [88, 89]. RDNI can also regulate the prostate cancer pathway by interacting with 7 targets. This pathway has connection with both inflammation-related targets and prostate cancer, indicating that RDNI may have potential therapeutic effect against prostate cancer [90]. Degree centralities, betweenness centralities, and closeness centralities of other pathways in Figures 2(a) and 2(b) are generally lower, but they are also important in the inflammatory process, such as IL-17 signaling pathway, NF-*κ*B signaling pathway, and arachidonic acid metabolism pathway. It is worth mentioning that these pathways are closely relevant to the former four pathways.

### 3.3. Cross-Talks among Inflammation-Related Pathways

**Figure 4:**
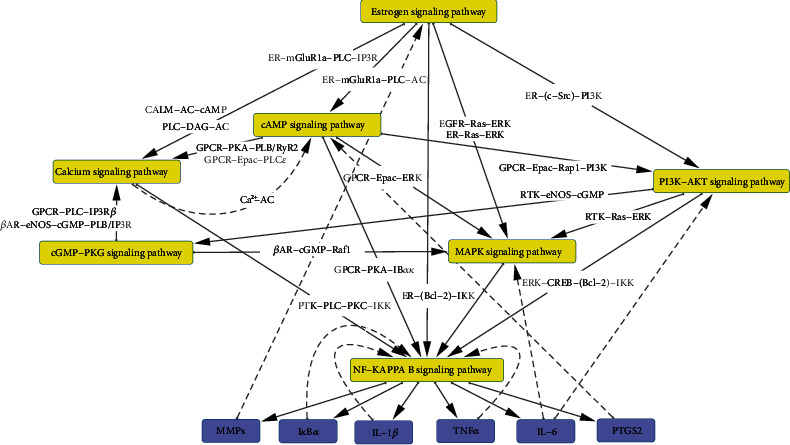
Cross-talks between seven primary regulatory pathways of RDNI for inflammation. Gray edge represents that the pathway in front of the arrow is regulated by the upstream pathway. The connection between two pathways is marked on the edge. The feedback regulations are marked as dashed lines.

Cross-talks among pathways are common in the regulation of biological processes. They are normally connected by key targets or common upstream/downstream pathways. The integration and correlation analysis of these pathways can help understand the MOA of TCM thoroughly and comprehensively. We analyzed the cross-talks within four key inflammation-related pathways regulated by RDNI, namely, estrogen signaling pathway, PI3K-AKT signaling pathway, cGMP-PKG signaling pathway, and calcium signaling pathway. Another three pathways were introduced to bridge the gaps among these key pathways, namely, cAMP signaling pathway (hsa04024), MAPK signaling pathway (hsa04010), and NF-*κ*B signaling pathway (hsa04064). The cross-talk network (Figure 4) shows that each pathway is directly related to the NF-*κ*B signaling pathway. The expression products of NF-*κ*B, such as IL-1*β* [91], TNF*α* [92], PTGS2, interleukin 6 (IL-6) [93], MMPs, and Bcl-2, play significant roles in the inflammatory process. Associations with NF-*κ*B signaling pathways suggest the importance of other pathways in the inflammatory process. The estrogen signaling pathway is located in the upstream of the whole regulatory network, and therefore, CAA can link almost all inflammatory processes in the downstream pathway by modulating ER. Meanwhile, ER can regulate the expression of BCL2 gene and then the expression of NF-*κ*B as well as inflammatory factors succinctly. The cAMP signaling pathway is the direct upstream of the PI3K-AKT signaling pathway, calcium signaling pathway, and NF-*κ*B signaling pathway; hence, CAA and its derivative FA can influence the downstream inflammatory process by regulating the formation of the cAMP.

The MAPK signaling pathway connects the estrogen signaling pathway, PI3K-AKT signaling pathway, cGMP-PKG signaling pathway, and cAMP signaling pathway to the expression of NF-*κ*B through the Ras pathway. In the calcium signaling pathway, the generation of Ca^2+^ is regulated by cAMP through multiple approaches. Ca^2+^ can regulate the expression of cAMP in the form of feedback by Ca^2+^-dependent adenylate cyclase (AC) and PKC*θ*. The generation of cGMP is regulated by NO whose concentrate is regulated by eNOS, a downstream enzyme of AKT. It can also interact with regulatory targets of Ca^2+^ just like cAMP. The expression product of NF-*κ*B such as IL-1*β*, TNF*α*, and I*κ*B*α* regulates both the expression of IKK and the dissociation of the I*κ*B*α*-NF-*κ*B complex. The generation of IL-6, PTGS2, and MMPs also has feedback regulation on upstream pathways. These feedback regulation and the mediation of the MAPK signaling pathway increase the complexity and robustness of the regulatory effects of RDNI on the inflammatory process.

## 4. Conclusions

In this work, the anti-inflammatory mechanism of RDNI was explored by network pharmacological methods. Eighty-four targets of RDNI were collected by data mining and molecular docking to construct a compound-target network. Key targets (Bcl-2, eNOS, PTGS2, PPARA, and MMPs) were found to be responsible for regulating the inflammatory process by RDNI compounds and metabolites. Two hundred and one pathways were found to be connected with RDNI targets. Four key pathways, namely, estrogen signaling pathway, PI3K-AKT signaling pathway, cGMP-PKG signaling pathway, and calcium signaling pathway, would play important roles. The cross-talks among four key pathways and another three related pathways were further identified. Results demonstrate that RDNI, an injection formed by multiple ingredients, can interact with multifarious inflammation-related targets. The interactions make RDNI capable of regulating multiple biological processes and treat inflammation at the systems level. Moreover, TCM is a complicated drug system; thus, complex interactions between multicomponents and multitargets make it possible to regulate multipathways and biological processes. Although the conclusions obtained in this research require to be verified by further experiments, network pharmacology provides a promising approach to investigate the MOA of TCM.

## Figures and Tables

**Table 1:** Ingredients and metabolites of RDNI.

Compound	Abbreviation	Content in injection (mg/ml)	MW	CID	CAS
Neochlorogenic acid	5CQA	2.51	354.31	5280633	906-33-2
Chlorogenic acid	CGA	4.36	354.31	1794427	327-97-9
Cryptochlorogenic acid	4CQA	2.01	354.31	9798666	905-99-7
Caffeic acid	CAA	0.09	180.15	689043	331-39-5
Isochlorogenic acid B	IsoB	0.33	516.45	5281780	14534-61-3
Isochlorogenic acid A	IsoA	0.19	516.45	6474310	89919-62-0
Isochlorogenic acid C	IsoC	0.34	516.45	6474309	57378-72-0
Geniposide	Gen	8.16	404.36	107848	169799-41-1
Secoxyloganin	Sec	0.81	404.36	162868	58822-47-2
Ferulic acid^∗^	FA	NA	194.18	445858	537-98-4
Dihydroferulic acid^∗^	DFA	NA	196.20	17865499	NA
Genipin^∗^	Gep	NA	226.22	442424	6902-77-8
3′-Hydroxycinnamic acid^∗^	NA	NA	164.16	637541	588-30-7

^∗^Ferulic acid, dihydroferulic acid, genipin, and 3′-hydroxycinnamic acid are metabolite of caffeic acid, caffeic acid, geniposide, and caffeic acid, respectively.

**Table 2:** Targets obtained by molecular docking.

Compound	Target	UniProt ID	Binding energy (kcal/mol)
4CQA	Progesterone receptor	P06401	-9.15
	5′-AMP-activated protein kinase subunit beta-2	O43741	-9.06
	Thiopurine S-methyltransferase	P51580	-8.98
	Complement C1q subcomponent subunit C	P02747	-8.51
	Histamine H1 receptor	P35367	-8.42
5CQA	Progesterone receptor	P06401	-9.05
	5′-AMP-activated protein kinase subunit beta-2	O43741	-8.69
	Phospholipase A2, membrane associated	P14555	-8.56
	Glutathione S-transferase A2	P09210	-8.4
	Retinoic acid receptor alpha	P10276	-8.38
CGA	Thiopurine S-methyltransferase	P51580	-8.74
	Androgen receptor	P10275	-8.32
	Phospholipase A2, membrane associated	P14555	-8.28
Gen	Thiopurine S-methyltransferase	P51580	-8.41
Gep	Corticosteroid 11-beta-dehydrogenase isozyme 1	P28845	-8.19
IsoA	Phospholipase A2, membrane associated	P14555	-11.82
	5′-AMP-activated protein kinase subunit beta-2	O43741	-10.32
	Thiopurine S-methyltransferase	P51580	-10.24
	Peroxisome proliferator-activated receptor gamma	P37231	-9.08
	Glucocorticoid receptor	P04150	-9.02
	Fatty acid-binding protein, intestinal	P12104	-8.82
	Aldose reductase	P15121	-8.66
	Histamine H1 receptor	P35367	-8.51
	Nitric oxide synthase, endothelial	P29474	-8.28
IsoB	Progesterone receptor	P06401	-10.62
	Thiopurine S-methyltransferase	P51580	-10.52
	Peroxisome proliferator-activated receptor gamma	P37231	-9.18
	Prostaglandin reductase 2	Q8N8N7	-8.91
	Peroxisome proliferator-activated receptor alpha	Q07869	-8.89
	Glucocorticoid receptor	P04150	-8.29
IsoC	5′-AMP-activated protein kinase subunit beta-2	O43741	-10.61
	Progesterone receptor	P06401	-10.4
	Phospholipase A2, membrane associated	P14555	-9.97
	Peroxisome proliferator-activated receptor gamma	P37231	-9.97
	Retinoic acid receptor beta	P10826	-9.46
	Thiopurine S-methyltransferase	P51580	-8.89
	Myeloperoxidase	P05164	-8.54
	Histamine H1 receptor	P35367	-8.29

## Data Availability

The data used to support the findings of this study are included within the supplementary information files.

## Supplementary Materials

Supplementary Materials Supplementary Table S1: targets gathered in PubChem, BindingDB, and TCMSP. Supplementary Table S2: network topological parameters of CTN. Supplementary Table S3: target-pathway relationships of potential targets of RDNI. Supplementary Table S4: network topological parameters of TPN. Supplementary Figure S1: HPLC spectra of RDNI at 237 nm (a) and 324 nm (b).
