# Supplementary material for: Assessing the Anti-inflammatory Mechanism of Reduning Injection by Network Pharmacology
Source: Biomed Res Int. 2020 Dec 16;2020:6134098. doi: 10.1155/2020/6134098 (PMC7758122; doi:10.1155/2020/6134098)
Supplement: Supplementary Materials — Supplementary Table S1: targets gathered in PubChem, BindingDB, and TCMSP. Supplementary Table S2: network topological parameters of CTN. Supplementary Table S3: target-pathway relationships of potential targets of RDNI. Supplementary Table S4: network topological parameters of TPN. Supplementary Figure S1: HPLC spectra of RDNI at 237 nm (a) and 324 nm (b). [file 6134098.f1.docx]

**Assessing the anti-inflammatory mechanism of Reduning Injection by network pharmacology**

Fuda Xie,^1,2^ Mingxiang Xie,^1,2^ Yibing Yang,^1,2^ Miaomiao Zhang,^3^ Xiaojie Xu,^4^ Na Liu,^1,2^ Wei Xiao,^5,*^ Jiangyong Gu^1,2*^

^1^Research Center of Integrative Medicine, School of Basic Medical Science, Guangzhou University of Chinese Medicine, Guangzhou 510006, China.

^2^Department of Biochemistry, School of Basic Medical Science, Guangzhou University of Chinese Medicine, Guangzhou 510006, China.

^3^The Second Clinical College, Guangzhou University of Chinese Medicine, Guangzhou 510006, China.

^4^College of Chemistry and Molecular Engineering, Peking University, Beijing 100871, China.

^5^State Key Laboratory of New-Tech for Chinese Medicine Pharmaceutical Process, Lianyungang 222001, China.

Correspondence should be addressed to Wei Xiao (xw_kanion@163.com) and Jiangyong Gu (gujy@gzucm.edu.cn).


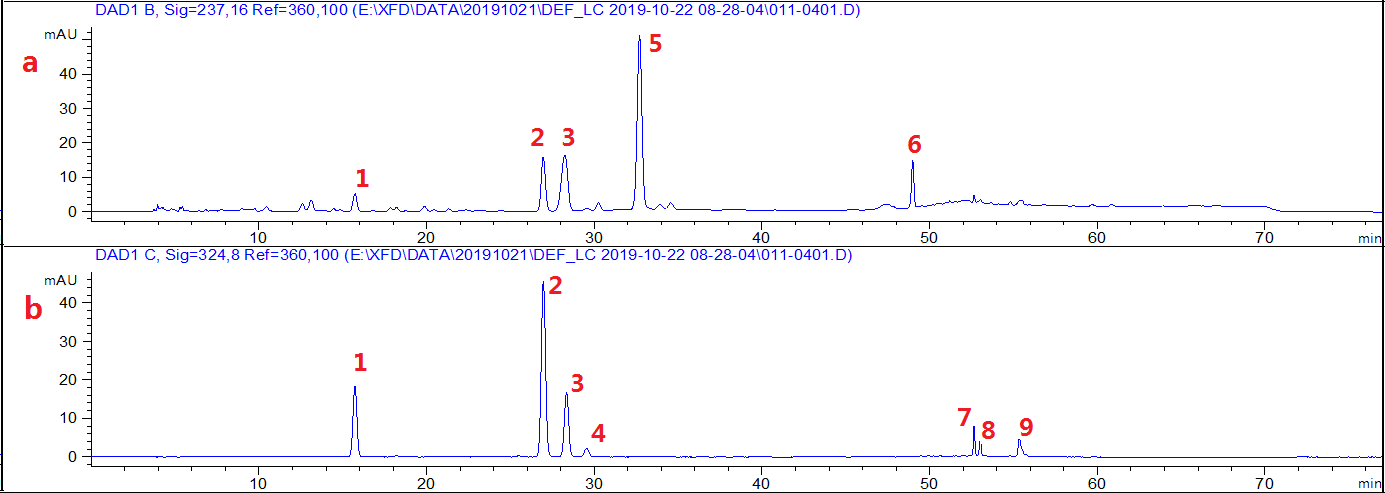


**Supplementary Figure S1:** HPLC spectra of RDNI at 237nm (a) and 324nm (b). The numbered peaks represent the nine compounds of RDNI: neochlorogenic acid (1), chlorogenic acid (2), cryptochlorogenic acid (3), caffeic acid (4), geniposide (5), secoxyloganin (6), isochlorogenic acid B (7), isochlorogenic acid A (8), isochlorogenic acid B (9).

| **Supplementary Table S1** Targets gathered in PubChem, Binding DB and TCMSP | | | |
| --- | --- | --- | --- |
| **Compound** | **Target** | **Abbreviation** | **Uniprot ID** |
| CAA | Aldose Reductase | AKR1B1 | P15121 |
|  | Arachidonate 5-lipoxygenase | ALOX5 | P09917 |
|  | Alkaline phosphatase, tissue-nonspecific isozyme | ALPL | P05186 |
|  | Carbonic Anhydrase 1 | CA1 | P00915 |
|  | Carbonic Anhydrase 12 | CA12 | O43570 |
|  | Carbonic Anhydrase 14 | CA14 | Q9ULX7 |
|  | Carbonic Anhydrase 2 | CA2 | P00918 |
|  | Carbonic Anhydrase 3 | CA3 | P07451 |
|  | Carbonic Anhydrase 4 | CA4 | P22748 |
|  | Carbonic Anhydrase 5A, Mitochondrial | CA5A | P35218 |
|  | Carbonic Anhydrase 5B, Mitochondrial | CA5B | Q9Y2D0 |
|  | Carbonic Anhydrase 6 | CA6 | P23280 |
|  | Carbonic Anhydrase 7 | CA7 | P43166 |
|  | Carbonic Anhydrase 9 | CA9 | Q16790 |
|  | Carbonic Anhydrase 13 | CA13 | Q8N1Q1 |
|  | Estrogen Receptor Beta | ESR2 | Q92731 |
|  | Lysosomal alpha-glucosidase | GAA | P10253 |
|  | Glyceraldehyde-3-phosphate dehydrogenase | GAPDH | P04406 |
|  | FAD-linked sulfhydryl oxidase ALR | GFER | P55789 |
|  | Glutaminase Kidney Isoform, Mitochondrial | GLS | O94925 |
|  | Heat shock protein HSP 90-alpha | HSP90AA1 | P07900 |
|  | Heat shock protein HSP 90-beta | HSP90AB1 | P08238 |
|  | Induced myeloid leukemia cell differentiation protein Mcl-1 | Mcl-1 | Q07820 |
|  | Interstitial Collagenase | MMP1 | P03956 |
|  | 72 kDa type IV collagenase | MMP2 | P08253 |
|  | Matrix metalloproteinase 9 | MMP9 | P14780 |
|  | Mannose-6-phosphate isomerase | MPI | P34949 |
|  | Prostaglandin G/H synthase 1 | PTGS1 | P23219 |
|  | Beta-1 adrenergic receptor | ADRB1 | P08588 |
|  | Prostaglandin G/H synthase 2 | PTGS2 | P35354 |
|  | Alpha-2A adrenergic receptor | ADRA2A | P08913 |
|  | Alpha-2C adrenergic receptor | ADRA2C | P18825 |
|  | Beta-2 adrenergic receptor | ADRB2 | P07550 |
|  | Amine oxidase [flavin-containing] B | MAOB | P27338 |
|  | Amine oxidase [flavin-containing] A | MAOA | P21397 |
|  | Epidermal growth factor receptor | EGFR | P00533 |
|  | Chymotrypsinogen B | CTRB1 | P17538 |
| FA | Arachidonate 5-lipoxygenase | ALOX15 | P09917 |
|  | Amyloid-beta A4 protein | APP | P05067 |
|  | Carbonic Anhydrase 1 | CA1 | P00915 |
|  | Carbonic Anhydrase 12 | CA12 | O43570 |
|  | Carbonic Anhydrase 14 | CA14 | Q9ULX7 |
|  | Carbonic Anhydrase 2 | CA2 | P00918 |
|  | Carbonic Anhydrase 3 | CA3 | P07451 |
|  | Carbonic Anhydrase 4 | CA4 | P22748 |
|  | Carbonic Anhydrase 5A, Mitochondrial | CA5A | P35218 |
|  | Carbonic Anhydrase 5B, Mitochondrial | CA5B | Q9Y2D0 |
|  | Carbonic Anhydrase 6 | CA6 | P23280 |
|  | Carbonic Anhydrase 7 | CA7 | P43166 |
|  | Carbonic Anhydrase 9 | CA9 | Q16790 |
|  | Carbonic Anhydrase 13 | Car13 | Q8N1Q1 |
|  | Frataxin, Mitochondrial | FXN | Q16595 |
|  | FAD-linked sulfhydryl oxidase ALR | GFER | P55789 |
|  | Prostaglandin G/H synthase 1 | PTGS1 | P23219 |
|  | Prostaglandin G/H synthase 2 | PTGS2 | P35354 |
|  | Nitric-oxide synthase, endothelial | eNOS | P29474 |
|  | Alpha-2A adrenergic receptor | ADRA2A | P08913 |
|  | Sodium-dependent noradrenaline transporter | SLC6A2 | P23975 |
|  | Alpha-1A adrenergic receptor | ADRA1A | P35348 |
|  | Sodium-dependent dopamine transporter | SLC6A3 | Q01959 |
|  | Beta-2 adrenergic receptor | ADRB2 | P07550 |
|  | Leukotriene A-4 hydrolase | LTA4H | P09960 |
|  | Amine oxidase [flavin-containing] B | MAOB | P27338 |
|  | Amine oxidase [flavin-containing] A | MAOA | P21397 |
|  | Chymotrypsinogen B | CTRB1 | P17538 |
|  | mRNA of PKA Catalytic Subunit C-alpha | PRKACA | P17612 |
|  | Alpha-2B adrenergic receptor | ADRA2B | P18089 |
|  | Urokinase-type plasminogen activator | PLAU | P00749 |
|  | Heat shock protein HSP 90 | HSP90AB1 | P08238 |
|  | β-Carbonic anhydrase 3 | CA3 | P07451 |
| Gen | Carbonic anhydrase II | CA2 | P00918 |
|  | Apoptosis regulator Bcl-2 | Bcl-2 | P10415 |
|  | Heme oxygenase 1 | HMOX1 | P09601 |
|  | Neuromodulin | GAP43 | P17677 |
|  | Phospholipase B1, membrane-associated | PLB1 | Q6P1J6 |
|  | Glucagon | GCG | P01275 |
|  | Glutathione S-transferase Mu 1 | GSTM1 | P09488 |
|  | Glutathione S-transferase Mu 2 | GSTM2 | P28161 |
| Gep | Prostaglandin G/H synthase 1 | PTGS1 | P23219 |
|  | Prostaglandin G/H synthase 2 | PTGS2 | P35354 |
|  | Carbonic anhydrase II | CA2 | P00918 |
|  | Gamma-aminobutyric-acid receptor alpha-2 subunit | GABRA2 | P47869 |
|  | Gamma-aminobutyric acid receptor subunit alpha-1 | GABRA1 | P14867 |
|  | Thymidine kinase | TK1 | P04183 |
|  | Trypsin-1 | PRSS1 | P07477 |
|  | Glutamate receptor 2 | GRIA2 | P42262 |
|  | Gamma-aminobutyric-acid receptor subunit alpha-6 | GABRA6 | Q16445 |
|  | Canalicular multispecific organic anion transporter 1 | ABCC2 | Q92887 |
|  | Early Growth Response Protein 1 | EGR1 | P18146 |
| IsoA | Aldose Reductase | AKR1B1 | P15121 |
|  | Aldo-keto reductase family 1 member B10 | AKR1B10 | O60218 |
|  | Amyloid-beta A4 protein | APP | P05067 |
|  | mRNA of Protein-tyrosine phosphatase, non-receptor type 1 | PTPN1 | P18031 |
|  | Coagulation factor Xa | F10 | P00742 |
| IsoB | Aldose Reductase | AKR1B1 | P15121 |
|  | Aldo-keto reductase family 1 member B10 | AKR1B10 | O60218 |
|  | Amyloid-beta A4 protein | APP | P05067 |
|  | mRNA of Protein-tyrosine phosphatase, non-receptor type 1 | PTPN1 | P18031 |
|  | Coagulation factor Xa | F10 | P00742 |
| IsoC | Amyloid-beta A4 protein | APP | P05067 |
| CGA | mRNA of Protein-tyrosine phosphatase, non-receptor type 1 | PTPN1 | P18031 |
|  | FAD-linked sulfhydryl oxidase ALR | GFER | P55789 |
|  | Integrin alpha-4 | ITGA4 | P13612 |
|  | Prostaglandin G/H synthase 2 | PTGS2 | P35354 |
| 5CQA | Lysosomal alpha-glucosidase | GAA | P10253 |
| 4CQA | Prostaglandin G/H synthase 2 | PTGS2 | P35354 |
| Sec | Carbonic anhydrase II | CA2 | P00918 |
|  | Dipeptidyl peptidase IV | DPP4 | P27487 |

**Supplementary Table S2** Network topological parameters of CTN

| **Target Uniprot ID** | **Degree centrality** | **Betweenness centrality** | **Closeness centrality** |
| --- | --- | --- | --- |
| P51580 | 6 | 0.07622685 | 0.32081911 |
| P00918 | 5 | 0.19148582 | 0.41409692 |
| P35354 | 5 | 0.1340029 | 0.41048035 |
| O43741 | 4 | 0.01360021 | 0.29283489 |
| P05067 | 4 | 0.08463165 | 0.35741445 |
| P06401 | 4 | 0.01554422 | 0.29283489 |
| P14555 | 4 | 0.01818677 | 0.29652997 |
| P15121 | 4 | 0.08478283 | 0.37450199 |
| P23219 | 3 | 0.04294203 | 0.37751004 |
| P35367 | 3 | 0.00427314 | 0.28571429 |
| P37231 | 3 | 0.0025207 | 0.26934097 |
| P55789 | 3 | 0.0185906 | 0.36293436 |
| O43570 | 2 | 0.0021602 | 0.34944238 |
| O60218 | 2 | 4.23E-04 | 0.26478873 |
| P00742 | 2 | 4.23E-04 | 0.26478873 |
| P00915 | 2 | 0.0021602 | 0.34944238 |
| P04150 | 2 | 4.23E-04 | 0.26478873 |
| P07451 | 2 | 0.0021602 | 0.34944238 |
| P07550 | 2 | 0.0021602 | 0.34944238 |
| P08238 | 2 | 0.0021602 | 0.34944238 |
| P08913 | 2 | 0.0021602 | 0.34944238 |
| P09917 | 2 | 0.0021602 | 0.34944238 |
| P10253 | 2 | 0.038722 | 0.33691756 |
| P17538 | 2 | 0.0021602 | 0.34944238 |
| P18031 | 2 | 4.23E-04 | 0.26478873 |
| P21397 | 2 | 0.0021602 | 0.34944238 |
| P22748 | 2 | 0.0021602 | 0.34944238 |
| P23280 | 2 | 0.0021602 | 0.34944238 |
| P27338 | 2 | 0.0021602 | 0.34944238 |
| P29474 | 2 | 0.02000543 | 0.33935018 |
| P35218 | 2 | 0.0021602 | 0.34944238 |
| P43166 | 2 | 0.0021602 | 0.34944238 |
| Q16790 | 2 | 0.0021602 | 0.34944238 |
| Q8N1Q1 | 2 | 0.0021602 | 0.34944238 |
| Q9ULX7 | 2 | 0.0021602 | 0.34944238 |
| Q9Y2D0 | 2 | 0.0021602 | 0.34944238 |
| O94925 | 1 | 0 | 0.31649832 |
| P00533 | 1 | 0 | 0.31649832 |
| P00749 | 1 | 0 | 0.30618893 |
| P01275 | 1 | 0 | 0.26628895 |
| P02747 | 1 | 0 | 0.26330532 |
| P03956 | 1 | 0 | 0.31649832 |
| P04183 | 1 | 0 | 0.25201072 |
| P04406 | 1 | 0 | 0.31649832 |
| P05164 | 1 | 0 | 0.23558897 |
| P05186 | 1 | 0 | 0.31649832 |
| P07477 | 1 | 0 | 0.25201072 |
| P07900 | 1 | 0 | 0.31649832 |
| P08253 | 1 | 0 | 0.31649832 |
| P08588 | 1 | 0 | 0.31649832 |
| P09210 | 1 | 0 | 0.22871046 |
| P09488 | 1 | 0 | 0.26628895 |
| P09601 | 1 | 0 | 0.26628895 |
| P09960 | 1 | 0 | 0.30618893 |
| P10275 | 1 | 0 | 0.26478873 |
| P10276 | 1 | 0 | 0.22871046 |
| P10415 | 1 | 0 | 0.26628895 |
| P10826 | 1 | 0 | 0.23558897 |
| P12104 | 1 | 0 | 0.25895317 |
| P13612 | 1 | 0 | 0.26478873 |
| P14780 | 1 | 0 | 0.31649832 |
| P14867 | 1 | 0 | 0.25201072 |
| P17612 | 1 | 0 | 0.30618893 |
| P17677 | 1 | 0 | 0.26628895 |
| P18089 | 1 | 0 | 0.30618893 |
| P18146 | 1 | 0 | 0.25201072 |
| P18825 | 1 | 0 | 0.31649832 |
| P23975 | 1 | 0 | 0.30618893 |
| P27487 | 1 | 0 | 0.22871046 |
| P28161 | 1 | 0 | 0.26628895 |
| P28845 | 1 | 0 | 0.25201072 |
| P34949 | 1 | 0 | 0.31649832 |
| P35348 | 1 | 0 | 0.30618893 |
| P42262 | 1 | 0 | 0.25201072 |
| P47869 | 1 | 0 | 0.25201072 |
| Q01959 | 1 | 0 | 0.30618893 |
| Q07820 | 1 | 0 | 0.31649832 |
| Q07869 | 1 | 0 | 0.25613079 |
| Q16445 | 1 | 0 | 0.25201072 |
| Q16595 | 1 | 0 | 0.30618893 |
| Q6P1J6 | 1 | 0 | 0.26628895 |
| Q8N8N7 | 1 | 0 | 0.25613079 |
| Q92731 | 1 | 0 | 0.31649832 |
| Q92887 | 1 | 0 | 0.25201072 |
| Q2VPJ6 | 1 | 0 | 0.31803279 |
| Q6P7A9 | 1 | 0 | 0.31803279 |

| **Supplementary Table S3** Target-pathway relationships of potential targets of RDNI | | | |
| --- | --- | --- | --- |
| **Target** | **Gene ID** | **Pathway** | **Pathway Name** |
| O43570 | 771 | hsa00910 | Nitrogen metabolism |
| O60218 | 57016 | hsa00040 | Pentose and glucuronate interconversions |
| O60218 | 57016 | hsa00051 | Fructose and mannose metabolism |
| O60218 | 57016 | hsa00052 | Galactose metabolism |
| O60218 | 57016 | hsa00561 | Glycerolipid metabolism |
| O60218 | 57016 | hsa00790 | Folate biosynthesis |
| O60218 | 57016 | hsa01100 | Metabolic pathways |
| O94925 | 2744 | hsa00220 | Arginine biosynthesis |
| O94925 | 2744 | hsa00250 | Alanine, aspartate and glutamate metabolism |
| O94925 | 2744 | hsa00471 | D-Glutamine and D-glutamate metabolism |
| O94925 | 2744 | hsa01100 | Metabolic pathways |
| O94925 | 2744 | hsa04724 | Glutamatergic synapse |
| O94925 | 2744 | hsa04727 | GABAergic synapse |
| O94925 | 2744 | hsa04964 | Proximal tubule bicarbonate reclamation |
| O94925 | 2744 | hsa05206 | MicroRNAs in cancer |
| O94925 | 2744 | hsa05230 | Central carbon metabolism in cancer |
| P00533 | 1956 | hsa01521 | EGFR tyrosine kinase inhibitor resistance |
| P00533 | 1956 | hsa01522 | Endocrine resistance |
| P00533 | 1956 | hsa04010 | MAPK signaling pathway |
| P00533 | 1956 | hsa04012 | ErbB signaling pathway |
| P00533 | 1956 | hsa04014 | Ras signaling pathway |
| P00533 | 1956 | hsa04015 | Rap1 signaling pathway |
| P00533 | 1956 | hsa04020 | Calcium signaling pathway |
| P00533 | 1956 | hsa04066 | HIF-1 signaling pathway |
| P00533 | 1956 | hsa04068 | FoxO signaling pathway |
| P00533 | 1956 | hsa04072 | Phospholipase D signaling pathway |
| P00533 | 1956 | hsa04144 | Endocytosis |
| P00533 | 1956 | hsa04151 | PI3K-Akt signaling pathway |
| P00533 | 1956 | hsa04510 | Focal adhesion |
| P00533 | 1956 | hsa04520 | Adherens junction |
| P00533 | 1956 | hsa04540 | Gap junction |
| P00533 | 1956 | hsa04630 | Jak-STAT signaling pathway |
| P00533 | 1956 | hsa04810 | Regulation of actin cytoskeleton |
| P00533 | 1956 | hsa04912 | GnRH signaling pathway |
| P00533 | 1956 | hsa04915 | Estrogen signaling pathway |
| P00533 | 1956 | hsa04921 | Oxytocin signaling pathway |
| P00533 | 1956 | hsa04926 | Relaxin signaling pathway |
| P00533 | 1956 | hsa04928 | Parathyroid hormone synthesis, secretion and action |
| P00533 | 1956 | hsa04934 | Cushing syndrome |
| P00533 | 1956 | hsa05120 | Epithelial cell signaling in Helicobacter pylori infection |
| P00533 | 1956 | hsa05160 | Hepatitis C |
| P00533 | 1956 | hsa05163 | Human cytomegalovirus infection |
| P00533 | 1956 | hsa05165 | Human papillomavirus infection |
| P00533 | 1956 | hsa05200 | Pathways in cancer |
| P00533 | 1956 | hsa05205 | Proteoglycans in cancer |
| P00533 | 1956 | hsa05206 | MicroRNAs in cancer |
| P00533 | 1956 | hsa05210 | Colorectal cancer |
| P00533 | 1956 | hsa05212 | Pancreatic cancer |
| P00533 | 1956 | hsa05213 | Endometrial cancer |
| P00533 | 1956 | hsa05214 | Glioma |
| P00533 | 1956 | hsa05215 | Prostate cancer |
| P00533 | 1956 | hsa05218 | Melanoma |
| P00533 | 1956 | hsa05219 | Bladder cancer |
| P00533 | 1956 | hsa05223 | Non-small cell lung cancer |
| P00533 | 1956 | hsa05224 | Breast cancer |
| P00533 | 1956 | hsa05225 | Hepatocellular carcinoma |
| P00533 | 1956 | hsa05226 | Gastric cancer |
| P00533 | 1956 | hsa05230 | Central carbon metabolism in cancer |
| P00533 | 1956 | hsa05231 | Choline metabolism in cancer |
| P00742 | 2159 | hsa04610 | Complement and coagulation cascades |
| P00749 | 5328 | hsa04064 | NF-kappa B signaling pathway |
| P00749 | 5328 | hsa04610 | Complement and coagulation cascades |
| P00749 | 5328 | hsa05202 | Transcriptional misregulation in cancer |
| P00749 | 5328 | hsa05205 | Proteoglycans in cancer |
| P00749 | 5328 | hsa05206 | MicroRNAs in cancer |
| P00749 | 5328 | hsa05215 | Prostate cancer |
| P00915 | 759 | hsa00910 | Nitrogen metabolism |
| P00918 | 760 | hsa00910 | Nitrogen metabolism |
| P00918 | 760 | hsa04964 | Proximal tubule bicarbonate reclamation |
| P00918 | 760 | hsa04966 | Collecting duct acid secretion |
| P00918 | 760 | hsa04971 | Gastric acid secretion |
| P00918 | 760 | hsa04972 | Pancreatic secretion |
| P00918 | 760 | hsa04976 | Bile secretion |
| P01275 | 2641 | hsa04714 | Thermogenesis |
| P01275 | 2641 | hsa04911 | Insulin secretion |
| P01275 | 2641 | hsa04922 | Glucagon signaling pathway |
| P03956 | 4312 | hsa03320 | PPAR signaling pathway |
| P03956 | 4312 | hsa04657 | IL-17 signaling pathway |
| P03956 | 4312 | hsa04926 | Relaxin signaling pathway |
| P03956 | 4312 | hsa05200 | Pathways in cancer |
| P03956 | 4312 | hsa05219 | Bladder cancer |
| P03956 | 4312 | hsa05323 | Rheumatoid arthritis |
| P04183 | 7083 | hsa00240 | Pyrimidine metabolism |
| P04183 | 7083 | hsa00983 | Drug metabolism - other enzymes |
| P04183 | 7083 | hsa01100 | Metabolic pathways |
| P04406 | 2597 | hsa00010 | Glycolysis / Gluconeogenesis |
| P04406 | 2597 | hsa01100 | Metabolic pathways |
| P04406 | 2597 | hsa01200 | Carbon metabolism |
| P04406 | 2597 | hsa01230 | Biosynthesis of amino acids |
| P04406 | 2597 | hsa04066 | HIF-1 signaling pathway |
| P04406 | 2597 | hsa05010 | Alzheimer disease |
| P05067 | 351 | hsa04726 | Serotonergic synapse |
| P05067 | 351 | hsa05010 | Alzheimer disease |
| P05186 | 249 | hsa00730 | Thiamine metabolism |
| P05186 | 249 | hsa00790 | Folate biosynthesis |
| P05186 | 249 | hsa01100 | Metabolic pathways |
| P07451 | 761 | hsa00910 | Nitrogen metabolism |
| P07477 | 5644 | hsa04080 | Neuroactive ligand-receptor interaction |
| P07477 | 5644 | hsa04972 | Pancreatic secretion |
| P07477 | 5644 | hsa04974 | Protein digestion and absorption |
| P07477 | 5644 | hsa05164 | Influenza A |
| P07550 | 154 | hsa04020 | Calcium signaling pathway |
| P07550 | 154 | hsa04022 | cGMP-PKG signaling pathway |
| P07550 | 154 | hsa04024 | cAMP signaling pathway |
| P07550 | 154 | hsa04080 | Neuroactive ligand-receptor interaction |
| P07550 | 154 | hsa04261 | Adrenergic signaling in cardiomyocytes |
| P07550 | 154 | hsa04923 | Regulation of lipolysis in adipocytes |
| P07550 | 154 | hsa04924 | Renin secretion |
| P07550 | 154 | hsa04970 | Salivary secretion |
| P07900 | 3320 | hsa04141 | Protein processing in endoplasmic reticulum |
| P07900 | 3320 | hsa04151 | PI3K-Akt signaling pathway |
| P07900 | 3320 | hsa04217 | Necroptosis |
| P07900 | 3320 | hsa04612 | Antigen processing and presentation |
| P07900 | 3320 | hsa04621 | NOD-like receptor signaling pathway |
| P07900 | 3320 | hsa04657 | IL-17 signaling pathway |
| P07900 | 3320 | hsa04659 | Th17 cell differentiation |
| P07900 | 3320 | hsa04914 | Progesterone-mediated oocyte maturation |
| P07900 | 3320 | hsa04915 | Estrogen signaling pathway |
| P07900 | 3320 | hsa05200 | Pathways in cancer |
| P07900 | 3320 | hsa05215 | Prostate cancer |
| P07900 | 3320 | hsa05418 | Fluid shear stress and atherosclerosis |
| P08238 | 3326 | hsa04141 | Protein processing in endoplasmic reticulum |
| P08238 | 3326 | hsa04151 | PI3K-Akt signaling pathway |
| P08238 | 3326 | hsa04217 | Necroptosis |
| P08238 | 3326 | hsa04612 | Antigen processing and presentation |
| P08238 | 3326 | hsa04621 | NOD-like receptor signaling pathway |
| P08238 | 3326 | hsa04657 | IL-17 signaling pathway |
| P08238 | 3326 | hsa04659 | Th17 cell differentiation |
| P08238 | 3326 | hsa04914 | Progesterone-mediated oocyte maturation |
| P08238 | 3326 | hsa04915 | Estrogen signaling pathway |
| P08238 | 3326 | hsa05200 | Pathways in cancer |
| P08238 | 3326 | hsa05215 | Prostate cancer |
| P08238 | 3326 | hsa05418 | Fluid shear stress and atherosclerosis |
| P08253 | 4313 | hsa01522 | Endocrine resistance |
| P08253 | 4313 | hsa04670 | Leukocyte transendothelial migration |
| P08253 | 4313 | hsa04912 | GnRH signaling pathway |
| P08253 | 4313 | hsa04915 | Estrogen signaling pathway |
| P08253 | 4313 | hsa04926 | Relaxin signaling pathway |
| P08253 | 4313 | hsa04933 | AGE-RAGE signaling pathway in diabetic complications |
| P08253 | 4313 | hsa05200 | Pathways in cancer |
| P08253 | 4313 | hsa05205 | Proteoglycans in cancer |
| P08253 | 4313 | hsa05219 | Bladder cancer |
| P08253 | 4313 | hsa05418 | Fluid shear stress and atherosclerosis |
| P08588 | 153 | hsa04020 | Calcium signaling pathway |
| P08588 | 153 | hsa04022 | cGMP-PKG signaling pathway |
| P08588 | 153 | hsa04024 | cAMP signaling pathway |
| P08588 | 153 | hsa04080 | Neuroactive ligand-receptor interaction |
| P08588 | 153 | hsa04261 | Adrenergic signaling in cardiomyocytes |
| P08588 | 153 | hsa04540 | Gap junction |
| P08588 | 153 | hsa04923 | Regulation of lipolysis in adipocytes |
| P08588 | 153 | hsa04924 | Renin secretion |
| P08588 | 153 | hsa04970 | Salivary secretion |
| P08588 | 153 | hsa05414 | Dilated cardiomyopathy (DCM) |
| P08913 | 150 | hsa04022 | cGMP-PKG signaling pathway |
| P08913 | 150 | hsa04080 | Neuroactive ligand-receptor interaction |
| P09488 | 2944 | hsa00480 | Glutathione metabolism |
| P09488 | 2944 | hsa00980 | Metabolism of xenobiotics by cytochrome P450 |
| P09488 | 2944 | hsa00982 | Drug metabolism - cytochrome P450 |
| P09488 | 2944 | hsa00983 | Drug metabolism - other enzymes |
| P09488 | 2944 | hsa01524 | Platinum drug resistance |
| P09488 | 2944 | hsa05200 | Pathways in cancer |
| P09488 | 2944 | hsa05204 | Chemical carcinogenesis |
| P09488 | 2944 | hsa05225 | Hepatocellular carcinoma |
| P09488 | 2944 | hsa05418 | Fluid shear stress and atherosclerosis |
| P09601 | 3162 | hsa00860 | Porphyrin and chlorophyll metabolism |
| P09601 | 3162 | hsa01100 | Metabolic pathways |
| P09601 | 3162 | hsa04066 | HIF-1 signaling pathway |
| P09601 | 3162 | hsa04216 | Ferroptosis |
| P09601 | 3162 | hsa04978 | Mineral absorption |
| P09601 | 3162 | hsa05200 | Pathways in cancer |
| P09601 | 3162 | hsa05206 | MicroRNAs in cancer |
| P09601 | 3162 | hsa05225 | Hepatocellular carcinoma |
| P09601 | 3162 | hsa05418 | Fluid shear stress and atherosclerosis |
| P09917 | 240 | hsa00590 | Arachidonic acid metabolism |
| P09917 | 240 | hsa01100 | Metabolic pathways |
| P09917 | 240 | hsa04664 | Fc epsilon RI signaling pathway |
| P09917 | 240 | hsa04726 | Serotonergic synapse |
| P09917 | 240 | hsa04913 | Ovarian steroidogenesis |
| P09917 | 240 | hsa05145 | Toxoplasmosis |
| P09960 | 4048 | hsa00590 | Arachidonic acid metabolism |
| P09960 | 4048 | hsa01100 | Metabolic pathways |
| P10253 | 2548 | hsa00052 | Galactose metabolism |
| P10253 | 2548 | hsa00500 | Starch and sucrose metabolism |
| P10253 | 2548 | hsa01100 | Metabolic pathways |
| P10253 | 2548 | hsa04142 | Lysosome |
| P10415 | 596 | hsa01521 | EGFR tyrosine kinase inhibitor resistance |
| P10415 | 596 | hsa01522 | Endocrine resistance |
| P10415 | 596 | hsa01524 | Platinum drug resistance |
| P10415 | 596 | hsa04064 | NF-kappa B signaling pathway |
| P10415 | 596 | hsa04066 | HIF-1 signaling pathway |
| P10415 | 596 | hsa04071 | Sphingolipid signaling pathway |
| P10415 | 596 | hsa04115 | p53 signaling pathway |
| P10415 | 596 | hsa04140 | Autophagy - animal |
| P10415 | 596 | hsa04141 | Protein processing in endoplasmic reticulum |
| P10415 | 596 | hsa04151 | PI3K-Akt signaling pathway |
| P10415 | 596 | hsa04210 | Apoptosis |
| P10415 | 596 | hsa04215 | Apoptosis - multiple species |
| P10415 | 596 | hsa04217 | Necroptosis |
| P10415 | 596 | hsa04261 | Adrenergic signaling in cardiomyocytes |
| P10415 | 596 | hsa04340 | Hedgehog signaling pathway |
| P10415 | 596 | hsa04510 | Focal adhesion |
| P10415 | 596 | hsa04621 | NOD-like receptor signaling pathway |
| P10415 | 596 | hsa04630 | Jak-STAT signaling pathway |
| P10415 | 596 | hsa04722 | Neurotrophin signaling pathway |
| P10415 | 596 | hsa04725 | Cholinergic synapse |
| P10415 | 596 | hsa04915 | Estrogen signaling pathway |
| P10415 | 596 | hsa04928 | Parathyroid hormone synthesis, secretion and action |
| P10415 | 596 | hsa04933 | AGE-RAGE signaling pathway in diabetic complications |
| P10415 | 596 | hsa05014 | Amyotrophic lateral sclerosis (ALS) |
| P10415 | 596 | hsa05145 | Toxoplasmosis |
| P10415 | 596 | hsa05152 | Tuberculosis |
| P10415 | 596 | hsa05161 | Hepatitis B |
| P10415 | 596 | hsa05169 | Epstein-Barr virus infection |
| P10415 | 596 | hsa05200 | Pathways in cancer |
| P10415 | 596 | hsa05206 | MicroRNAs in cancer |
| P10415 | 596 | hsa05210 | Colorectal cancer |
| P10415 | 596 | hsa05215 | Prostate cancer |
| P10415 | 596 | hsa05222 | Small cell lung cancer |
| P10415 | 596 | hsa05226 | Gastric cancer |
| P10415 | 596 | hsa05418 | Fluid shear stress and atherosclerosis |
| P13612 | 3676 | hsa04151 | PI3K-Akt signaling pathway |
| P13612 | 3676 | hsa04510 | Focal adhesion |
| P13612 | 3676 | hsa04512 | ECM-receptor interaction |
| P13612 | 3676 | hsa04514 | Cell adhesion molecules (CAMs) |
| P13612 | 3676 | hsa04640 | Hematopoietic cell lineage |
| P13612 | 3676 | hsa04670 | Leukocyte transendothelial migration |
| P13612 | 3676 | hsa04672 | Intestinal immune network for IgA production |
| P13612 | 3676 | hsa04810 | Regulation of actin cytoskeleton |
| P13612 | 3676 | hsa05140 | Leishmaniasis |
| P13612 | 3676 | hsa05165 | Human papillomavirus infection |
| P13612 | 3676 | hsa05410 | Hypertrophic cardiomyopathy (HCM) |
| P13612 | 3676 | hsa05412 | Arrhythmogenic right ventricular cardiomyopathy (ARVC) |
| P13612 | 3676 | hsa05414 | Dilated cardiomyopathy (DCM) |
| P14780 | 4318 | hsa01522 | Endocrine resistance |
| P14780 | 4318 | hsa04657 | IL-17 signaling pathway |
| P14780 | 4318 | hsa04668 | TNF signaling pathway |
| P14780 | 4318 | hsa04670 | Leukocyte transendothelial migration |
| P14780 | 4318 | hsa04915 | Estrogen signaling pathway |
| P14780 | 4318 | hsa04926 | Relaxin signaling pathway |
| P14780 | 4318 | hsa05161 | Hepatitis B |
| P14780 | 4318 | hsa05200 | Pathways in cancer |
| P14780 | 4318 | hsa05202 | Transcriptional misregulation in cancer |
| P14780 | 4318 | hsa05205 | Proteoglycans in cancer |
| P14780 | 4318 | hsa05206 | MicroRNAs in cancer |
| P14780 | 4318 | hsa05215 | Prostate cancer |
| P14780 | 4318 | hsa05219 | Bladder cancer |
| P14780 | 4318 | hsa05418 | Fluid shear stress and atherosclerosis |
| P14867 | 2554 | hsa04080 | Neuroactive ligand-receptor interaction |
| P14867 | 2554 | hsa04723 | Retrograde endocannabinoid signaling |
| P14867 | 2554 | hsa04727 | GABAergic synapse |
| P14867 | 2554 | hsa04742 | Taste transduction |
| P14867 | 2554 | hsa05032 | Morphine addiction |
| P14867 | 2554 | hsa05033 | Nicotine addiction |
| P15121 | 231 | hsa00040 | Pentose and glucuronate interconversions |
| P15121 | 231 | hsa00051 | Fructose and mannose metabolism |
| P15121 | 231 | hsa00052 | Galactose metabolism |
| P15121 | 231 | hsa00561 | Glycerolipid metabolism |
| P15121 | 231 | hsa00790 | Folate biosynthesis |
| P15121 | 231 | hsa01100 | Metabolic pathways |
| P17538 | 1504 | hsa04972 | Pancreatic secretion |
| P17538 | 1504 | hsa04974 | Protein digestion and absorption |
| P17612 | 5566 | hsa01522 | Endocrine resistance |
| P17612 | 5566 | hsa04010 | MAPK signaling pathway |
| P17612 | 5566 | hsa04014 | Ras signaling pathway |
| P17612 | 5566 | hsa04020 | Calcium signaling pathway |
| P17612 | 5566 | hsa04024 | cAMP signaling pathway |
| P17612 | 5566 | hsa04062 | Chemokine signaling pathway |
| P17612 | 5566 | hsa04114 | Oocyte meiosis |
| P17612 | 5566 | hsa04140 | Autophagy - animal |
| P17612 | 5566 | hsa04211 | Longevity regulating pathway |
| P17612 | 5566 | hsa04213 | Longevity regulating pathway - multiple species |
| P17612 | 5566 | hsa04261 | Adrenergic signaling in cardiomyocytes |
| P17612 | 5566 | hsa04270 | Vascular smooth muscle contraction |
| P17612 | 5566 | hsa04310 | Wnt signaling pathway |
| P17612 | 5566 | hsa04340 | Hedgehog signaling pathway |
| P17612 | 5566 | hsa04371 | Apelin signaling pathway |
| P17612 | 5566 | hsa04530 | Tight junction |
| P17612 | 5566 | hsa04540 | Gap junction |
| P17612 | 5566 | hsa04611 | Platelet activation |
| P17612 | 5566 | hsa04713 | Circadian entrainment |
| P17612 | 5566 | hsa04714 | Thermogenesis |
| P17612 | 5566 | hsa04720 | Long-term potentiation |
| P17612 | 5566 | hsa04723 | Retrograde endocannabinoid signaling |
| P17612 | 5566 | hsa04724 | Glutamatergic synapse |
| P17612 | 5566 | hsa04725 | Cholinergic synapse |
| P17612 | 5566 | hsa04726 | Serotonergic synapse |
| P17612 | 5566 | hsa04727 | GABAergic synapse |
| P17612 | 5566 | hsa04728 | Dopaminergic synapse |
| P17612 | 5566 | hsa04740 | Olfactory transduction |
| P17612 | 5566 | hsa04742 | Taste transduction |
| P17612 | 5566 | hsa04750 | Inflammatory mediator regulation of TRP channels |
| P17612 | 5566 | hsa04910 | Insulin signaling pathway |
| P17612 | 5566 | hsa04911 | Insulin secretion |
| P17612 | 5566 | hsa04912 | GnRH signaling pathway |
| P17612 | 5566 | hsa04913 | Ovarian steroidogenesis |
| P17612 | 5566 | hsa04914 | Progesterone-mediated oocyte maturation |
| P17612 | 5566 | hsa04915 | Estrogen signaling pathway |
| P17612 | 5566 | hsa04916 | Melanogenesis |
| P17612 | 5566 | hsa04918 | Thyroid hormone synthesis |
| P17612 | 5566 | hsa04919 | Thyroid hormone signaling pathway |
| P17612 | 5566 | hsa04921 | Oxytocin signaling pathway |
| P17612 | 5566 | hsa04922 | Glucagon signaling pathway |
| P17612 | 5566 | hsa04923 | Regulation of lipolysis in adipocytes |
| P17612 | 5566 | hsa04924 | Renin secretion |
| P17612 | 5566 | hsa04925 | Aldosterone synthesis and secretion |
| P17612 | 5566 | hsa04926 | Relaxin signaling pathway |
| P17612 | 5566 | hsa04927 | Cortisol synthesis and secretion |
| P17612 | 5566 | hsa04928 | Parathyroid hormone synthesis, secretion and action |
| P17612 | 5566 | hsa04934 | Cushing syndrome |
| P17612 | 5566 | hsa04961 | Endocrine and other factor-regulated calcium reabsorption |
| P17612 | 5566 | hsa04962 | Vasopressin-regulated water reabsorption |
| P17612 | 5566 | hsa04970 | Salivary secretion |
| P17612 | 5566 | hsa04971 | Gastric acid secretion |
| P17612 | 5566 | hsa04976 | Bile secretion |
| P17612 | 5566 | hsa05012 | Parkinson disease |
| P17612 | 5566 | hsa05020 | Prion diseases |
| P17612 | 5566 | hsa05030 | Cocaine addiction |
| P17612 | 5566 | hsa05031 | Amphetamine addiction |
| P17612 | 5566 | hsa05032 | Morphine addiction |
| P17612 | 5566 | hsa05034 | Alcoholism |
| P17612 | 5566 | hsa05110 | Vibrio cholerae infection |
| P17612 | 5566 | hsa05146 | Amoebiasis |
| P17612 | 5566 | hsa05163 | Human cytomegalovirus infection |
| P17612 | 5566 | hsa05165 | Human papillomavirus infection |
| P17612 | 5566 | hsa05166 | HTLV-I infection |
| P17612 | 5566 | hsa05169 | Epstein-Barr virus infection |
| P17612 | 5566 | hsa05200 | Pathways in cancer |
| P17612 | 5566 | hsa05203 | Viral carcinogenesis |
| P17612 | 5566 | hsa05205 | Proteoglycans in cancer |
| P17612 | 5566 | hsa05414 | Dilated cardiomyopathy (DCM) |
| P18031 | 5770 | hsa04520 | Adherens junction |
| P18031 | 5770 | hsa04910 | Insulin signaling pathway |
| P18031 | 5770 | hsa04931 | Insulin resistance |
| P18089 | 151 | hsa04022 | cGMP-PKG signaling pathway |
| P18089 | 151 | hsa04080 | Neuroactive ligand-receptor interaction |
| P18146 | 1958 | hsa04371 | Apelin signaling pathway |
| P18146 | 1958 | hsa04912 | GnRH signaling pathway |
| P18146 | 1958 | hsa04928 | Parathyroid hormone synthesis, secretion and action |
| P18146 | 1958 | hsa04933 | AGE-RAGE signaling pathway in diabetic complications |
| P18146 | 1958 | hsa05020 | Prion diseases |
| P18146 | 1958 | hsa05166 | HTLV-I infection |
| P18825 | 152 | hsa04022 | cGMP-PKG signaling pathway |
| P18825 | 152 | hsa04080 | Neuroactive ligand-receptor interaction |
| P21397 | 4128 | hsa00260 | Glycine, serine and threonine metabolism |
| P21397 | 4128 | hsa00330 | Arginine and proline metabolism |
| P21397 | 4128 | hsa00340 | Histidine metabolism |
| P21397 | 4128 | hsa00350 | Tyrosine metabolism |
| P21397 | 4128 | hsa00360 | Phenylalanine metabolism |
| P21397 | 4128 | hsa00380 | Tryptophan metabolism |
| P21397 | 4128 | hsa00982 | Drug metabolism - cytochrome P450 |
| P21397 | 4128 | hsa01100 | Metabolic pathways |
| P21397 | 4128 | hsa04726 | Serotonergic synapse |
| P21397 | 4128 | hsa04728 | Dopaminergic synapse |
| P21397 | 4128 | hsa05030 | Cocaine addiction |
| P21397 | 4128 | hsa05031 | Amphetamine addiction |
| P21397 | 4128 | hsa05034 | Alcoholism |
| P22748 | 762 | hsa00910 | Nitrogen metabolism |
| P22748 | 762 | hsa04964 | Proximal tubule bicarbonate reclamation |
| P23219 | 5742 | hsa00590 | Arachidonic acid metabolism |
| P23219 | 5742 | hsa01100 | Metabolic pathways |
| P23219 | 5742 | hsa04611 | Platelet activation |
| P23219 | 5742 | hsa04726 | Serotonergic synapse |
| P23219 | 5742 | hsa04923 | Regulation of lipolysis in adipocytes |
| P23280 | 765 | hsa00910 | Nitrogen metabolism |
| P27338 | 4129 | hsa00260 | Glycine, serine and threonine metabolism |
| P27338 | 4129 | hsa00330 | Arginine and proline metabolism |
| P27338 | 4129 | hsa00340 | Histidine metabolism |
| P27338 | 4129 | hsa00350 | Tyrosine metabolism |
| P27338 | 4129 | hsa00360 | Phenylalanine metabolism |
| P27338 | 4129 | hsa00380 | Tryptophan metabolism |
| P27338 | 4129 | hsa00982 | Drug metabolism - cytochrome P450 |
| P27338 | 4129 | hsa01100 | Metabolic pathways |
| P27338 | 4129 | hsa04726 | Serotonergic synapse |
| P27338 | 4129 | hsa04728 | Dopaminergic synapse |
| P27338 | 4129 | hsa05030 | Cocaine addiction |
| P27338 | 4129 | hsa05031 | Amphetamine addiction |
| P27338 | 4129 | hsa05034 | Alcoholism |
| P27487 | 1803 | hsa04974 | Protein digestion and absorption |
| P28161 | 2946 | hsa00480 | Glutathione metabolism |
| P28161 | 2946 | hsa00980 | Metabolism of xenobiotics by cytochrome P450 |
| P28161 | 2946 | hsa00982 | Drug metabolism - cytochrome P450 |
| P28161 | 2946 | hsa00983 | Drug metabolism - other enzymes |
| P28161 | 2946 | hsa01524 | Platinum drug resistance |
| P28161 | 2946 | hsa05200 | Pathways in cancer |
| P28161 | 2946 | hsa05204 | Chemical carcinogenesis |
| P28161 | 2946 | hsa05225 | Hepatocellular carcinoma |
| P28161 | 2946 | hsa05418 | Fluid shear stress and atherosclerosis |
| P29474 | 4846 | hsa00220 | Arginine biosynthesis |
| P29474 | 4846 | hsa00330 | Arginine and proline metabolism |
| P29474 | 4846 | hsa01100 | Metabolic pathways |
| P29474 | 4846 | hsa04020 | Calcium signaling pathway |
| P29474 | 4846 | hsa04022 | cGMP-PKG signaling pathway |
| P29474 | 4846 | hsa04066 | HIF-1 signaling pathway |
| P29474 | 4846 | hsa04071 | Sphingolipid signaling pathway |
| P29474 | 4846 | hsa04151 | PI3K-Akt signaling pathway |
| P29474 | 4846 | hsa04370 | VEGF signaling pathway |
| P29474 | 4846 | hsa04371 | Apelin signaling pathway |
| P29474 | 4846 | hsa04611 | Platelet activation |
| P29474 | 4846 | hsa04915 | Estrogen signaling pathway |
| P29474 | 4846 | hsa04921 | Oxytocin signaling pathway |
| P29474 | 4846 | hsa04926 | Relaxin signaling pathway |
| P29474 | 4846 | hsa04931 | Insulin resistance |
| P29474 | 4846 | hsa04933 | AGE-RAGE signaling pathway in diabetic complications |
| P29474 | 4846 | hsa05418 | Fluid shear stress and atherosclerosis |
| P34949 | 4351 | hsa00051 | Fructose and mannose metabolism |
| P34949 | 4351 | hsa00520 | Amino sugar and nucleotide sugar metabolism |
| P34949 | 4351 | hsa01100 | Metabolic pathways |
| P35218 | 763 | hsa00910 | Nitrogen metabolism |
| P35348 | 148 | hsa04020 | Calcium signaling pathway |
| P35348 | 148 | hsa04022 | cGMP-PKG signaling pathway |
| P35348 | 148 | hsa04080 | Neuroactive ligand-receptor interaction |
| P35348 | 148 | hsa04152 | AMPK signaling pathway |
| P35348 | 148 | hsa04261 | Adrenergic signaling in cardiomyocytes |
| P35348 | 148 | hsa04270 | Vascular smooth muscle contraction |
| P35348 | 148 | hsa04970 | Salivary secretion |
| P35354 | 5743 | hsa00590 | Arachidonic acid metabolism |
| P35354 | 5743 | hsa01100 | Metabolic pathways |
| P35354 | 5743 | hsa04064 | NF-kappa B signaling pathway |
| P35354 | 5743 | hsa04370 | VEGF signaling pathway |
| P35354 | 5743 | hsa04625 | C-type lectin receptor signaling pathway |
| P35354 | 5743 | hsa04657 | IL-17 signaling pathway |
| P35354 | 5743 | hsa04668 | TNF signaling pathway |
| P35354 | 5743 | hsa04723 | Retrograde endocannabinoid signaling |
| P35354 | 5743 | hsa04726 | Serotonergic synapse |
| P35354 | 5743 | hsa04913 | Ovarian steroidogenesis |
| P35354 | 5743 | hsa04921 | Oxytocin signaling pathway |
| P35354 | 5743 | hsa04923 | Regulation of lipolysis in adipocytes |
| P35354 | 5743 | hsa05140 | Leishmaniasis |
| P35354 | 5743 | hsa05163 | Human cytomegalovirus infection |
| P35354 | 5743 | hsa05165 | Human papillomavirus infection |
| P35354 | 5743 | hsa05167 | Kaposi sarcoma-associated herpesvirus infection |
| P35354 | 5743 | hsa05200 | Pathways in cancer |
| P35354 | 5743 | hsa05204 | Chemical carcinogenesis |
| P35354 | 5743 | hsa05206 | MicroRNAs in cancer |
| P35354 | 5743 | hsa05222 | Small cell lung cancer |
| P42262 | 2891 | hsa04024 | cAMP signaling pathway |
| P42262 | 2891 | hsa04080 | Neuroactive ligand-receptor interaction |
| P42262 | 2891 | hsa04713 | Circadian entrainment |
| P42262 | 2891 | hsa04720 | Long-term potentiation |
| P42262 | 2891 | hsa04723 | Retrograde endocannabinoid signaling |
| P42262 | 2891 | hsa04724 | Glutamatergic synapse |
| P42262 | 2891 | hsa04728 | Dopaminergic synapse |
| P42262 | 2891 | hsa04730 | Long-term depression |
| P42262 | 2891 | hsa05014 | Amyotrophic lateral sclerosis (ALS) |
| P42262 | 2891 | hsa05030 | Cocaine addiction |
| P42262 | 2891 | hsa05031 | Amphetamine addiction |
| P42262 | 2891 | hsa05033 | Nicotine addiction |
| P43166 | 766 | hsa00910 | Nitrogen metabolism |
| P47869 | 2555 | hsa04080 | Neuroactive ligand-receptor interaction |
| P47869 | 2555 | hsa04723 | Retrograde endocannabinoid signaling |
| P47869 | 2555 | hsa04727 | GABAergic synapse |
| P47869 | 2555 | hsa04742 | Taste transduction |
| P47869 | 2555 | hsa05032 | Morphine addiction |
| P47869 | 2555 | hsa05033 | Nicotine addiction |
| Q01959 | 6531 | hsa04728 | Dopaminergic synapse |
| Q01959 | 6531 | hsa05012 | Parkinson disease |
| Q01959 | 6531 | hsa05030 | Cocaine addiction |
| Q01959 | 6531 | hsa05031 | Amphetamine addiction |
| Q01959 | 6531 | hsa05034 | Alcoholism |
| Q07820 | 4170 | hsa04151 | PI3K-Akt signaling pathway |
| Q07820 | 4170 | hsa04210 | Apoptosis |
| Q07820 | 4170 | hsa04630 | Jak-STAT signaling pathway |
| Q07820 | 4170 | hsa05206 | MicroRNAs in cancer |
| Q16445 | 2559 | hsa04080 | Neuroactive ligand-receptor interaction |
| Q16445 | 2559 | hsa04723 | Retrograde endocannabinoid signaling |
| Q16445 | 2559 | hsa04727 | GABAergic synapse |
| Q16445 | 2559 | hsa04742 | Taste transduction |
| Q16445 | 2559 | hsa05032 | Morphine addiction |
| Q16445 | 2559 | hsa05033 | Nicotine addiction |
| Q16595 | 2395 | hsa00860 | Porphyrin and chlorophyll metabolism |
| Q16790 | 768 | hsa00910 | Nitrogen metabolism |
| Q6P1J6 | 151056 | hsa00564 | Glycerophospholipid metabolism |
| Q6P1J6 | 151056 | hsa00565 | Ether lipid metabolism |
| Q6P1J6 | 151056 | hsa00590 | Arachidonic acid metabolism |
| Q6P1J6 | 151056 | hsa00591 | Linoleic acid metabolism |
| Q6P1J6 | 151056 | hsa00592 | alpha-Linolenic acid metabolism |
| Q6P1J6 | 151056 | hsa01100 | Metabolic pathways |
| Q6P1J6 | 151056 | hsa04977 | Vitamin digestion and absorption |
| Q8N1Q1 | 377677 | hsa00910 | Nitrogen metabolism |
| Q92731 | 2100 | hsa01522 | Endocrine resistance |
| Q92731 | 2100 | hsa04915 | Estrogen signaling pathway |
| Q92731 | 2100 | hsa04917 | Prolactin signaling pathway |
| Q92731 | 2100 | hsa05200 | Pathways in cancer |
| Q92731 | 2100 | hsa05224 | Breast cancer |
| Q92887 | 1244 | hsa01523 | Antifolate resistance |
| Q92887 | 1244 | hsa01524 | Platinum drug resistance |
| Q92887 | 1244 | hsa02010 | ABC transporters |
| Q92887 | 1244 | hsa04976 | Bile secretion |
| Q9ULX7 | 23632 | hsa00910 | Nitrogen metabolism |
| Q9Y2D0 | 11238 | hsa00910 | Nitrogen metabolism |
| O43741 | 5565 | hsa04068 | FoxO signaling pathway |
| O43741 | 5565 | hsa04152 | AMPK signaling pathway |
| O43741 | 5565 | hsa04211 | Longevity regulating pathway |
| O43741 | 5565 | hsa04213 | Longevity regulating pathway - multiple species |
| O43741 | 5565 | hsa04371 | Apelin signaling pathway |
| O43741 | 5565 | hsa04530 | Tight junction |
| O43741 | 5565 | hsa04710 | Circadian rhythm |
| O43741 | 5565 | hsa04714 | Thermogenesis |
| O43741 | 5565 | hsa04910 | Insulin signaling pathway |
| O43741 | 5565 | hsa04920 | Adipocytokine signaling pathway |
| O43741 | 5565 | hsa04921 | Oxytocin signaling pathway |
| O43741 | 5565 | hsa04922 | Glucagon signaling pathway |
| O43741 | 5565 | hsa04931 | Insulin resistance |
| O43741 | 5565 | hsa04932 | Non-alcoholic fatty liver disease (NAFLD) |
| O43741 | 5565 | hsa05410 | Hypertrophic cardiomyopathy (HCM) |
| P02747 | 714 | hsa04610 | Complement and coagulation cascades |
| P02747 | 714 | hsa05020 | Prion diseases |
| P02747 | 714 | hsa05133 | Pertussis |
| P02747 | 714 | hsa05142 | Chagas disease (American trypanosomiasis) |
| P02747 | 714 | hsa05150 | Staphylococcus aureus infection |
| P02747 | 714 | hsa05322 | Systemic lupus erythematosus |
| P04150 | 2908 | hsa04080 | Neuroactive ligand-receptor interaction |
| P05164 | 4353 | hsa00983 | Drug metabolism - other enzymes |
| P05164 | 4353 | hsa04145 | Phagosome |
| P05164 | 4353 | hsa05202 | Transcriptional misregulation in cancer |
| P05164 | 4353 | hsa05221 | Acute myeloid leukemia |
| P06401 | 5241 | hsa04114 | Oocyte meiosis |
| P06401 | 5241 | hsa04914 | Progesterone-mediated oocyte maturation |
| P06401 | 5241 | hsa04915 | Estrogen signaling pathway |
| P06401 | 5241 | hsa05224 | Breast cancer |
| P09210 | 2939 | hsa00480 | Glutathione metabolism |
| P09210 | 2939 | hsa00980 | Metabolism of xenobiotics by cytochrome P450 |
| P09210 | 2939 | hsa00982 | Drug metabolism - cytochrome P450 |
| P09210 | 2939 | hsa00983 | Drug metabolism - other enzymes |
| P09210 | 2939 | hsa01524 | Platinum drug resistance |
| P09210 | 2939 | hsa05200 | Pathways in cancer |
| P09210 | 2939 | hsa05204 | Chemical carcinogenesis |
| P09210 | 2939 | hsa05225 | Hepatocellular carcinoma |
| P09210 | 2939 | hsa05418 | Fluid shear stress and atherosclerosis |
| P10275 | 367 | hsa04114 | Oocyte meiosis |
| P10275 | 367 | hsa05200 | Pathways in cancer |
| P10275 | 367 | hsa05215 | Prostate cancer |
| P10276 | 5914 | hsa04659 | Th17 cell differentiation |
| P10276 | 5914 | hsa04915 | Estrogen signaling pathway |
| P10276 | 5914 | hsa05200 | Pathways in cancer |
| P10276 | 5914 | hsa05202 | Transcriptional misregulation in cancer |
| P10276 | 5914 | hsa05221 | Acute myeloid leukemia |
| P10826 | 5915 | hsa05200 | Pathways in cancer |
| P10826 | 5915 | hsa05222 | Small cell lung cancer |
| P10826 | 5915 | hsa05223 | Non-small cell lung cancer |
| P10826 | 5915 | hsa05226 | Gastric cancer |
| P12104 | 2169 | hsa03320 | PPAR signaling pathway |
| P12104 | 2169 | hsa04975 | Fat digestion and absorption |
| P14555 | 5320 | hsa00564 | Glycerophospholipid metabolism |
| P14555 | 5320 | hsa00565 | Ether lipid metabolism |
| P14555 | 5320 | hsa00590 | Arachidonic acid metabolism |
| P14555 | 5320 | hsa00591 | Linoleic acid metabolism |
| P14555 | 5320 | hsa00592 | alpha-Linolenic acid metabolism |
| P14555 | 5320 | hsa01100 | Metabolic pathways |
| P14555 | 5320 | hsa04014 | Ras signaling pathway |
| P14555 | 5320 | hsa04270 | Vascular smooth muscle contraction |
| P14555 | 5320 | hsa04972 | Pancreatic secretion |
| P14555 | 5320 | hsa04975 | Fat digestion and absorption |
| P28845 | 3290 | hsa00140 | Steroid hormone biosynthesis |
| P28845 | 3290 | hsa00980 | Metabolism of xenobiotics by cytochrome P450 |
| P28845 | 3290 | hsa01100 | Metabolic pathways |
| P28845 | 3290 | hsa05204 | Chemical carcinogenesis |
| P35367 | 3269 | hsa04020 | Calcium signaling pathway |
| P35367 | 3269 | hsa04080 | Neuroactive ligand-receptor interaction |
| P35367 | 3269 | hsa04750 | Inflammatory mediator regulation of TRP channels |
| P37231 | 5468 | hsa03320 | PPAR signaling pathway |
| P37231 | 5468 | hsa04152 | AMPK signaling pathway |
| P37231 | 5468 | hsa04211 | Longevity regulating pathway |
| P37231 | 5468 | hsa04380 | Osteoclast differentiation |
| P37231 | 5468 | hsa04714 | Thermogenesis |
| P37231 | 5468 | hsa05016 | Huntington disease |
| P37231 | 5468 | hsa05200 | Pathways in cancer |
| P37231 | 5468 | hsa05202 | Transcriptional misregulation in cancer |
| P37231 | 5468 | hsa05216 | Thyroid cancer |
| P51580 | 7172 | hsa00983 | Drug metabolism - other enzymes |
| Q07869 | 5465 | hsa03320 | PPAR signaling pathway |
| Q07869 | 5465 | hsa04024 | cAMP signaling pathway |
| Q07869 | 5465 | hsa04920 | Adipocytokine signaling pathway |
| Q07869 | 5465 | hsa04922 | Glucagon signaling pathway |
| Q07869 | 5465 | hsa04931 | Insulin resistance |
| Q07869 | 5465 | hsa04932 | Non-alcoholic fatty liver disease (NAFLD) |
| Q07869 | 5465 | hsa05160 | Hepatitis C |

**Supplementary Table S4** Network topological parameters of TPN

| **Pathway** | **Degree centrality** | **Betweenness centrality** | **Closeness centrality** |
| --- | --- | --- | --- |
| hsa01100 | 19 | 0.18798299 | 0.31746032 |
| hsa05200 | 18 | 0.12650172 | 0.36553525 |
| hsa04080 | 13 | 0.03855147 | 0.24647887 |
| hsa00910 | 12 | 0.07123806 | 0.20588235 |
| hsa04915 | 11 | 0.03844984 | 0.34567901 |
| hsa05418 | 10 | 0.01768946 | 0.28629857 |
| hsa05206 | 8 | 0.03186583 | 0.31250000 |
| hsa04020 | 7 | 0.02303251 | 0.32941176 |
| hsa04022 | 7 | 0.01502908 | 0.26119403 |
| hsa04151 | 7 | 0.01822555 | 0.29350105 |
| hsa04726 | 7 | 0.02763004 | 0.31111111 |
| hsa05215 | 7 | 0.00767779 | 0.27290448 |
| hsa00590 | 6 | 0.00909243 | 0.26974952 |
| hsa00983 | 6 | 0.01561949 | 0.24390244 |
| hsa01522 | 6 | 0.01306800 | 0.31602709 |
| hsa04723 | 6 | 0.01585435 | 0.31180401 |
| hsa04926 | 6 | 0.01534986 | 0.33096927 |
| hsa00982 | 5 | 0.00523432 | 0.24822695 |
| hsa01524 | 5 | 0.01203620 | 0.26022305 |
| hsa04024 | 5 | 0.00863596 | 0.29106029 |
| hsa04261 | 5 | 0.01213514 | 0.30567686 |
| hsa04657 | 5 | 0.00377479 | 0.26515152 |
| hsa04727 | 5 | 0.01253052 | 0.30701754 |
| hsa04728 | 5 | 0.01144450 | 0.30769231 |
| hsa04921 | 5 | 0.01882105 | 0.33254157 |
| hsa04923 | 5 | 0.00975924 | 0.30973451 |
| hsa05030 | 5 | 0.01144450 | 0.30769231 |
| hsa05031 | 5 | 0.01144450 | 0.30769231 |
| hsa05202 | 5 | 0.01071563 | 0.24096386 |
| hsa05204 | 5 | 0.00535532 | 0.26022305 |
| hsa05205 | 5 | 0.01229075 | 0.30769231 |
| hsa05225 | 5 | 0.00670957 | 0.27343750 |
| hsa00980 | 4 | 0.00161697 | 0.24054983 |
| hsa03320 | 4 | 0.00651751 | 0.23411371 |
| hsa04066 | 4 | 0.01771337 | 0.28340081 |
| hsa04371 | 4 | 0.00724074 | 0.30701754 |
| hsa04714 | 4 | 0.00968338 | 0.28806584 |
| hsa04742 | 4 | 0.00459086 | 0.28513238 |
| hsa04912 | 4 | 0.00545440 | 0.30107527 |
| hsa04914 | 4 | 0.00555605 | 0.28571429 |
| hsa04922 | 4 | 0.00719309 | 0.28513238 |
| hsa04928 | 4 | 0.00893706 | 0.30973451 |
| hsa04931 | 4 | 0.00549440 | 0.25925926 |
| hsa04933 | 4 | 0.00342284 | 0.27613412 |
| hsa04970 | 4 | 0.00302498 | 0.28571429 |
| hsa04972 | 4 | 0.02730240 | 0.24734982 |
| hsa05032 | 4 | 0.00459086 | 0.28513238 |
| hsa05033 | 4 | 0.00030347 | 0.21604938 |
| hsa05034 | 4 | 0.00800151 | 0.29914530 |
| hsa05165 | 4 | 0.01994374 | 0.32558140 |
| hsa05219 | 4 | 0.00178333 | 0.25454545 |
| hsa05222 | 4 | 0.00606938 | 0.28806584 |
| hsa00051 | 3 | 0.00014764 | 0.19662921 |
| hsa00052 | 3 | 0.00021932 | 0.19690577 |
| hsa00330 | 3 | 0.00202180 | 0.25500911 |
| hsa00480 | 3 | 0.00000853 | 0.22328549 |
| hsa00790 | 3 | 0.00014764 | 0.19662921 |
| hsa04014 | 3 | 0.02092750 | 0.32258065 |
| hsa04064 | 3 | 0.00408603 | 0.27667984 |
| hsa04068 | 3 | 0.00497677 | 0.27667984 |
| hsa04114 | 3 | 0.00264028 | 0.28000000 |
| hsa04141 | 3 | 0.00039904 | 0.24390244 |
| hsa04152 | 3 | 0.00382706 | 0.24432810 |
| hsa04211 | 3 | 0.00692102 | 0.28747433 |
| hsa04217 | 3 | 0.00039904 | 0.24390244 |
| hsa04270 | 3 | 0.01172790 | 0.30701754 |
| hsa04510 | 3 | 0.00525562 | 0.26666667 |
| hsa04540 | 3 | 0.00531957 | 0.30501089 |
| hsa04610 | 3 | 0.01089573 | 0.21772939 |
| hsa04611 | 3 | 0.00422433 | 0.30368764 |
| hsa04621 | 3 | 0.00039904 | 0.24390244 |
| hsa04630 | 3 | 0.00167362 | 0.26217228 |
| hsa04659 | 3 | 0.00020503 | 0.22400000 |
| hsa04670 | 3 | 0.00224367 | 0.23569024 |
| hsa04724 | 3 | 0.00727289 | 0.30701754 |
| hsa04910 | 3 | 0.00558714 | 0.28340081 |
| hsa04913 | 3 | 0.00653433 | 0.30303030 |
| hsa04924 | 3 | 0.00171761 | 0.28455285 |
| hsa04964 | 3 | 0.01893682 | 0.24734982 |
| hsa04974 | 3 | 0.00752670 | 0.18087855 |
| hsa04976 | 3 | 0.04729186 | 0.29227557 |
| hsa05020 | 3 | 0.03431189 | 0.28282828 |
| hsa05163 | 3 | 0.00678719 | 0.31963470 |
| hsa05224 | 3 | 0.00117236 | 0.25000000 |
| hsa05226 | 3 | 0.00204817 | 0.26266417 |
| hsa05414 | 3 | 0.01097589 | 0.29166667 |
| hsa00040 | 2 | 0.00000427 | 0.19607843 |
| hsa00220 | 2 | 0.00107408 | 0.26119403 |
| hsa00260 | 2 | 0.00000197 | 0.22012579 |
| hsa00340 | 2 | 0.00000197 | 0.22012579 |
| hsa00350 | 2 | 0.00000197 | 0.22012579 |
| hsa00360 | 2 | 0.00000197 | 0.22012579 |
| hsa00380 | 2 | 0.00000197 | 0.22012579 |
| hsa00561 | 2 | 0.00000427 | 0.19607843 |
| hsa00564 | 2 | 0.00032027 | 0.23140496 |
| hsa00565 | 2 | 0.00032027 | 0.23140496 |
| hsa00591 | 2 | 0.00032027 | 0.23140496 |
| hsa00592 | 2 | 0.00032027 | 0.23140496 |
| hsa00860 | 2 | 0.00714286 | 0.23850085 |
| hsa01521 | 2 | 0.00072026 | 0.26168224 |
| hsa04010 | 2 | 0.00284661 | 0.29787234 |
| hsa04071 | 2 | 0.00121510 | 0.26923077 |
| hsa04140 | 2 | 0.00317116 | 0.29661017 |
| hsa04210 | 2 | 0.00035554 | 0.24179620 |
| hsa04213 | 2 | 0.00191654 | 0.28225806 |
| hsa04340 | 2 | 0.00317116 | 0.29661017 |
| hsa04370 | 2 | 0.00045062 | 0.26315789 |
| hsa04520 | 2 | 0.00189759 | 0.24911032 |
| hsa04530 | 2 | 0.00191654 | 0.28225806 |
| hsa04612 | 2 | 0.00000213 | 0.22222222 |
| hsa04668 | 2 | 0.00048159 | 0.25830258 |
| hsa04713 | 2 | 0.00137976 | 0.28513238 |
| hsa04720 | 2 | 0.00137976 | 0.28513238 |
| hsa04725 | 2 | 0.00317116 | 0.29661017 |
| hsa04750 | 2 | 0.00185872 | 0.28340081 |
| hsa04810 | 2 | 0.00205465 | 0.25225225 |
| hsa04911 | 2 | 0.00201071 | 0.27833002 |
| hsa04920 | 2 | 0.00015557 | 0.21705426 |
| hsa04932 | 2 | 0.00015557 | 0.21705426 |
| hsa04934 | 2 | 0.00284661 | 0.29787234 |
| hsa04971 | 2 | 0.03299252 | 0.28985507 |
| hsa04975 | 2 | 0.00303409 | 0.23529412 |
| hsa05010 | 2 | 0.00042144 | 0.22580645 |
| hsa05012 | 2 | 0.00101116 | 0.27833002 |
| hsa05014 | 2 | 0.00380296 | 0.25782689 |
| hsa05140 | 2 | 0.00339416 | 0.25735294 |
| hsa05145 | 2 | 0.00248634 | 0.26119403 |
| hsa05160 | 2 | 0.00241001 | 0.25316456 |
| hsa05161 | 2 | 0.00027450 | 0.24561404 |
| hsa05166 | 2 | 0.00070847 | 0.27888446 |
| hsa05169 | 2 | 0.00317116 | 0.29661017 |
| hsa05210 | 2 | 0.00072026 | 0.26168224 |
| hsa05221 | 2 | 0.00093175 | 0.21943574 |
| hsa05230 | 2 | 0.00328929 | 0.27667984 |
| hsa05410 | 2 | 0.00185173 | 0.22400000 |
| hsa00010 | 1 | 0 | 0.21052632 |
| hsa00140 | 1 | 0 | 0.19746121 |
| hsa00240 | 1 | 0 | 0.19858156 |
| hsa00250 | 1 | 0 | 0.23688663 |
| hsa00471 | 1 | 0 | 0.23688663 |
| hsa00500 | 1 | 0 | 0.19525802 |
| hsa00520 | 1 | 0 | 0.19498607 |
| hsa00730 | 1 | 0 | 0.19498607 |
| hsa01200 | 1 | 0 | 0.21052632 |
| hsa01230 | 1 | 0 | 0.21052632 |
| hsa01523 | 1 | 0 | 0.19774011 |
| hsa02010 | 1 | 0 | 0.19774011 |
| hsa04012 | 1 | 0 | 0.24778761 |
| hsa04015 | 1 | 0 | 0.24778761 |
| hsa04062 | 1 | 0 | 0.27777778 |
| hsa04072 | 1 | 0 | 0.24778761 |
| hsa04115 | 1 | 0 | 0.24137931 |
| hsa04142 | 1 | 0 | 0.19525802 |
| hsa04144 | 1 | 0 | 0.24778761 |
| hsa04145 | 1 | 0 | 0.17500000 |
| hsa04215 | 1 | 0 | 0.24137931 |
| hsa04216 | 1 | 0 | 0.23809524 |
| hsa04310 | 1 | 0 | 0.27777778 |
| hsa04380 | 1 | 0 | 0.22292994 |
| hsa04512 | 1 | 0 | 0.21505376 |
| hsa04514 | 1 | 0 | 0.21505376 |
| hsa04625 | 1 | 0 | 0.25179856 |
| hsa04640 | 1 | 0 | 0.21505376 |
| hsa04664 | 1 | 0 | 0.21943574 |
| hsa04672 | 1 | 0 | 0.21505376 |
| hsa04710 | 1 | 0 | 0.21244310 |
| hsa04722 | 1 | 0 | 0.24137931 |
| hsa04730 | 1 | 0 | 0.21406728 |
| hsa04740 | 1 | 0 | 0.27777778 |
| hsa04916 | 1 | 0 | 0.27777778 |
| hsa04917 | 1 | 0 | 0.21571649 |
| hsa04918 | 1 | 0 | 0.27777778 |
| hsa04919 | 1 | 0 | 0.27777778 |
| hsa04925 | 1 | 0 | 0.27777778 |
| hsa04927 | 1 | 0 | 0.27777778 |
| hsa04961 | 1 | 0 | 0.27777778 |
| hsa04962 | 1 | 0 | 0.27777778 |
| hsa04966 | 1 | 0 | 0.20260492 |
| hsa04977 | 1 | 0 | 0.19607843 |
| hsa04978 | 1 | 0 | 0.23809524 |
| hsa05016 | 1 | 0 | 0.22292994 |
| hsa05110 | 1 | 0 | 0.27777778 |
| hsa05120 | 1 | 0 | 0.24778761 |
| hsa05133 | 1 | 0 | 0.18421053 |
| hsa05142 | 1 | 0 | 0.18421053 |
| hsa05146 | 1 | 0 | 0.27777778 |
| hsa05150 | 1 | 0 | 0.18421053 |
| hsa05152 | 1 | 0 | 0.24137931 |
| hsa05164 | 1 | 0 | 0.18041237 |
| hsa05167 | 1 | 0 | 0.25179856 |
| hsa05203 | 1 | 0 | 0.27777778 |
| hsa05212 | 1 | 0 | 0.24778761 |
| hsa05213 | 1 | 0 | 0.24778761 |
| hsa05214 | 1 | 0 | 0.24778761 |
| hsa05216 | 1 | 0 | 0.22292994 |
| hsa05218 | 1 | 0 | 0.24778761 |
| hsa05223 | 1 | 0 | 0.21244310 |
| hsa05231 | 1 | 0 | 0.24778761 |
| hsa05322 | 1 | 0 | 0.18421053 |
| hsa05323 | 1 | 0 | 0.21739130 |
| hsa05412 | 1 | 0 | 0.21505376 |
